# Adaptive Recombinant Nanoworms from Genetically Encodable Star Amphiphiles

**DOI:** 10.1021/acs.biomac.1c01314

**Published:** 2021-12-23

**Authors:** Md Shahadat Hossain, Jingjing Ji, Christopher J. Lynch, Miguel Guzman, Shikha Nangia, Davoud Mozhdehi

**Affiliations:** † Department of Chemistry, Syracuse University, 1-014 Center for Science and Technology, 111 College Place, Syracuse, New York 13244, United States; ‡ Department of Biomedical and Chemical Engineering, Syracuse University, 329 Link Hall, Syracuse, New York 13244, United States; § BioInspired Syracuse: Institute for Material and Living Systems, Syracuse University, Syracuse, New York 13244, United States

## Abstract

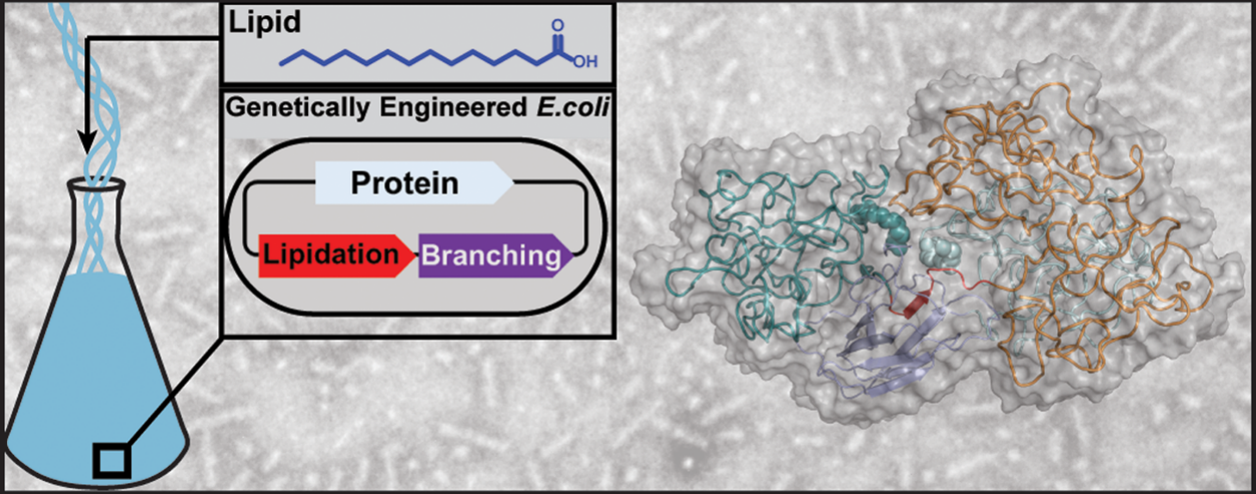

Recombinant nanoworms are promising candidates for materials and biomedical applications ranging from the templated synthesis of nanomaterials to multivalent display of bioactive peptides and targeted delivery of theranostic agents. However, molecular design principles to synthesize these assemblies (which are thermodynamically favorable only in a narrow region of the phase diagram) remain unclear. To advance the identification of design principles for the programmable assembly of proteins into well-defined nanoworms and to broaden their stability regimes, we were inspired by the ability of topologically engineered synthetic macromolecules to acess rare mesophases. To test this design principle in biomacromolecular assemblies, we used post-translational modifications (PTMs) to generate lipidated proteins with precise topological and compositional asymmetry. Using an integrated experimental and computational approach, we show that the material properties (thermoresponse and nanoscale assembly) of these hybrid amphiphiles are modulated by their amphiphilic architecture. Importantly, we demonstrate that the judicious choice of amphiphilic architecture can be used to program the assembly of proteins into adaptive nanoworms, which undergo a morphological transition (sphere-to-nanoworms) in response to temperature stimuli.

## Introduction

Nanoencapsulation of therapeutics and imaging agents can dramatically improve their efficacy and specificity while reducing their undesirable side effects.^1−3^ However, as the use of nanomaterials in medicine expands, new concerns regarding their off-target accumulation and toxicity have emerged.^4,5^ Nanobiomaterials such as proteins are promising platforms to address these concerns because, in addition to degradability, their sequence, structure, and function can be controlled with precision to modulate the carriers’ characteristics such as targeting,^6−8^ stealth,^9−11^ and immunomodulation,^12,13^ among others.^14−17^ Consequently, the precise engineering of the size and morphology of protein-based nanomaterials remains a key objective of the field as these characteristics regulate the pharmacokinetics and biodistribution of the encapsulated cargo.^7,18−22^ Specifically, rods are receiving increased attention because the higher aspect ratios of these anisotropic nanoparticles can increase cellular internalization and interaction with cell-surface receptors.^23−26^ Despite these promising attributes, molecular design rules to create protein-based rods with both radius and length below 200 nm (also known as nanoworms) remain unclear.

The rational design of nanoworms requires delicate optimization of building blocks’ “conformational asymmetry” because these assemblies are thermodynamically favorable only in a narrow range of the phase diagram.^27−31^ The conformational asymmetry of macromolecules can be adjusted by altering their amphiphilic composition and/or topology.^32,33^ However, because proteins are only expressed as a linear sequence of amino acids, the design of protein-based nanorods has exclusively relied on constructs with extreme compositional asymmetry.^34^ For instance, the MacKay and Chilkoti groups have designed nanoworms by fusing large, disordered elastinlike polypeptides (ELPs) to short dissimilar domains such as single-chain variable domain fragments^35,36^ or aromatic-rich peptides.^37,38^ However, the complex and nonintuitive dependence of nanoworms’s properties on protein sequence and features limits the widespread utility of this linear amphiphilic architecture. This is because small perturbations in composition or changes to solution parameters can result in polydisperse mixtures of cylindrical assemblies whose lengths range from nano- to micrometer. These difficulties in synthesis may hinder applications such as drug delivery or templated synthesis of nanomaterials, in which dispersity alters performance metrics such as biodistribution, endocytosis, and other desired functions of nanomaterials.^39^

To address these challenges and to create a new class of protein-based nanoworms for biomedical applications, we were inspired by a molecular design strategy from the world of synthetic polymers. It is well-established that changing the topology of block copolymers from linear to asymmetric stars, in which multiple hydrophobic and hydrophilic arms are covalently connected at a common junction, is effective for enhancing conformational asymmetry and accessing rare mesophases.^40−42^ Similarly, we envisioned that topological engineering of proteins could facilitate access to unique assemblies such as nanoworms by modulating the stability boundaries of these phases.

As a proof-of-principle, we focused on the simplest nonlinear topology: the miktoarm star in which two hydrophobic arms are compositionally identical while the third hydrophilic arm differs, i.e., A_2_B. To manipulate the protein’s topology (e.g., branching), we used the isopeptide ligation between split-protein pairs, SpyCatcher and SpyTag.^43^ This strategy has been used to synthesize the proteins of complex nonlinear topologies^44−46^ with enhanced stability and proteolytic resistance or for appending bioactive motifs to protein nanostructures.^47−49^ However, to the best of our knowledge, controlling the nanoassembly of proteins by topological engineering has not been reported. We reasoned that the progress has been limited because topological engineering alone may not provide the energetic driving force to compensate for the entropic penalty of self-organization. To overcome this barrier and induce nanoassembly, we combined topological engineering with lipidation PTM to generate hybrid protein amphiphiles with topological and compositional asymmetry.^50^

In this paper, the arms of the star (A or B) are based on a model thermoresponsive ELP with the canonical sequence of (GXGVP)_n_, whose composition is distinguished by the identity of the guest residue (X) and arm (n) length.^51−53^ The N-termini of hydrophobic arms were modified with a myristoyl group (C14:0) to generate star-shaped amphiphilic fatty acid-modified elastinlike polypeptides (SAFE). The amphiphilic architecture of SAFEs is defined by the hierarchical combination of the star topological asymmetry (compositional differences between the arms) and the pattern of lipidation (i.e., number and location).^54^ We hypothesized that the inter- and intra-arm interactions and the hydration of the arms could be modulated by changing the pattern of lipidation and/or the solution temperature, thus providing a dial to regulate the nanoassembly of SAFEs into nanoworms.

Here, we present the molecular design of the first generation of SAFEs and use scattering and microscopy to demonstrate that their material properties (assembly and thermoresponse) are modulated by their lipidation pattern. Using molecular dynamics simulations and principal component analysis, we reveal that the lipidation pattern influences the shape, size, and hydration of SAFE chains at the molecular level and that the changes in these microscopic features parallel observed trends in macroscopic properties as a function of lipidation pattern.

## Materials and Methods

### Materials

All materials were purchased from commercial sources and used as received without further purification. The complete list of chemicals and reagents—and their commercial suppliers—is provided in the Supporting Information.

### Cloning

Genes encoding linear building blocks V_40_-Tag-S_60_ and V_40_-Catcher (see the text for nomenclature) were constructed using Gibson assembly and recursive directional ligation by plasmid reconstruction. The identity of each gene was confirmed using Sanger sequencing. Additional details are provided in the Supporting Information.

### Protein Expression and Purification

Proteins were expressed in *Escherichia coli* BL21(DE3) grown in 2x YT medium under the control of lac promotor. To express myristoylated proteins, the growth media was supplemented with myristic acid (100 μM). All proteins were first purified by exploiting the lower critical solubility behavior of ELP followed by reversed-phase HPLC to ensure> 95% purity before self-assembly studies. Additional details are provided in the Supporting Information.

### Synthesis of Star Amphiphiles

Miktoarm star amphiphiles were synthesized by mixing the corresponding linear building blocks (ELP block copolymer and ELP-Catcher fusions) in reaction buffer (PBS or PBS supplemented with 4 M urea) and incubation at room temperature for 2 h. For instance, MMC was synthesized by reacting M-V_40_-Tag-S_60_ (30 μM) with M-V_40_-Catcher (20 μM). Reaction progress was monitored using SDS-PAGE and the appearance of the product band (∼75–100 kDa) and reduction in the intensity of starting material bands (∼50 and ∼37 kDa), Figure S2. Star amphiphiles were subsequently purified to homogeneity using RP-HPLC.

### Reversed-Phase High-Performance Liquid Chromatography (RP-HPLC)

Analytical and preparative RP-HPLC was performed on a Shimadzu instrument equipped with a photodiode array detector on C18 columns (Phenomenex Jupiter 5 μm C18 300 Å, 250 × 4.6, and 250 × 10 mm^2^). The mobile phase was a linear gradient of acetonitrile and water (0–90% acetonitrile over 40 min, each phase supplemented with 0.1% TFA).

### MALDI-TOF-MS

Matrix-assisted laser desorption/ionization, time-of-flight mass spectrometry (MALDI-TOF-MS) was conducted on a Bruker Autoflex III. N-terminal peptide fragments were characterized after digestion with trypsin.

### Circular Dichroism

The spectra were recorded on Aviv Model 420 CD spectrometer at 20, 30, 40, 50, and 65 °C and processed using Aviv software v3.47. Proteins were analyzed with a 1 mm path length quartz cell in 190–250 nm wavelength range. To minimize the spectroscopic interference from chloride ions, protein solutions were prepared in 10 mM phosphate buffer (pH 7.4) to the final concentration of 3.25 μM. The background spectrum recorded at 15 °C was subtracted from each spectrum before converting the data to mean molar residue ellipticity (MRE) in deg·cm^2^·mol^–1^. The data was deconvoluted by Dichroweb using CONTIN model.^55^

### Turbidimetry Assay

The thermal behavior of proteins was characterized using a Cary 100 UV–vis spectrophotometer (Agilent, Santa Clara, CA) equipped with a Peltier temperature controller. The optical density of the solution at 350 nm was recorded at 15–65 °C while heating the solution at the rate of 1 °C/min. For reversibility studies (Figure S18), protein solutions were subsequently cooled to 15 °C at a rate of 1 °C/min.

### Dynamic Light Scattering (DLS)

DLS was performed on a Zetasizer Nano (Malvern Instruments, U.K.) with a 173° backscatter detector. Before analysis, protein solutions (20 μM in PBS) were subject to centrifugation (21 000*g*, 5 min, 4 °C); supernatants were loaded into a DLS cuvette and analyzed at 15–65 °C (in 5 °C increments). The sample was incubated at each temperature for a minimum of 2 min to stabilize the temperature. Measurements were performed in triplicate at each temperature. Scattering autocorrelation functions were analyzed with Zetasizer software using the cumulant and CONTIN methods to calculate the hydrodynamic radii (*Z*_avg_), polydispersity index, and intensity-size distributions. For reversibility studies (Figure 4b), the mean scattering intensity was recorded at 20 °C (below *T*_t_) and subsequently at 50 °C (above *T*_t_) without changing the attenuator index. The protein solutions were then cooled to 20 °C again, and the scattering intensity was monitored for 250 min (at 30 min interval) without adjusting the attenuator settings.

### Transmission Electron Microscopy (TEM)

TEM imaging was performed using FEI Tecnai 12 BioTwin (Thermo Fisher Scientific, Waltham, MA) operated at 120 kV, equipped with Gatan SC1000A CCD camera. Protein solution (10 μL) was deposited onto a carbon-coated grid. After blotting excess solution, the grid was stained with 1% uranyl acetate for 1 min and air-dried at room temperature for 12 h before imaging.

### Cryo-TEM

Protein solution (4 μL, 20 μM in PBS) was deposited onto a freshly plasma-cleaned Quantifoil grid (Quantifoil Micro Tools GmbH, Germany), stored inside an environmentally controlled chamber, Mk IV Vitrobot (Thermo Fisher Scientific), with 100% humidity. After blotting the excess solution, the sample was vitrified by plunging the grid into liquid ethane. Grids were stored under liquid nitrogen until they were imaged on a Tecnai BioTwin 120 kV transmission electron microscope equipped with a Gatan SC1000A CCD camera, operated at liquid N_2_ temperature. Imaging was performed under low-dose conditions using a Gatan 626 or a Gatan 910 holder.

### Differential Interference Contrast Microscopy (DIC)

DIC was conducted on a Zeiss AxioObserver Z1 widefield microscope (Carl Zeiss Inc., Berlin, Germany), with an ORCA-Flash4.0 LT+ Digital CMOS camera (Hamamatsu Photonics, Hamamatsu, Japan). Images were analyzed using MetaMorph imaging software (Molecular Devices, CA). Protein solution in PBS was heated to 60 °C and applied onto a glass slide (10 μL), shielded with a coverslip, and imaged immediately.

### Molecular Dynamics (MD) Simulations

The atomistic structure of the SpyTag/SpyCatcher complex was obtained from the Protein Data Bank (PDB: 4MLI). The atomistic structures of disordered peptides (GVGVP)_40_ and (GSGVP)_60_ and the RS (GLYASKLFSNL) were obtained from I-TASSER (Iterative Threading ASSEmbly Refinement) server.^56−58^ YASARA was used to fuse peptide arms to the SpyTag/SpyCatcher.^59^ The systems were subjected to energy minimization and equilibration steps with the input files generated from CHARMM-GUI solution builder,^60−63^ where the N-termini of NNC were modified by myristic acids to generate MMC, MNC, and NMC systems. The CHARMM36m force field parameters were used for disordered protein,^64^ salt (0.14 M NaCl and 0.01 M Na_3_PO_4_), and explicit TIP3P water.^65^ All atomistic molecular dynamics simulations were carried out using the GROMACS version 2019.^66^ Each system was energy minimized, followed by equilibration in isothermal–isochoric (NVT) and isothermal–isobaric (NPT) for 1 ns each, and production MD run under NPT conditions for 500 ns. The heavy atoms of the disordered protein were restrained during NVT and NPT equilibration. All restraints were removed during the production MD. The temperature of each system was maintained at 37 °C using the velocity-rescale thermostat with τ_t_ = 1.0 ps.^67^ In the NPT equilibration step, isotropic pressure of 1 bar was maintained using Berendsen barostat with τ_p_ = 5.0 ps and compressibility of 4.5 × 10^–5^ bar^–1^.^68^ In the production MD, we used the Parrinello–Rahman barostat^69^ with τ_p_ = 5.0 ps and compressibility of 4.5 × 10^–5^ bar^–1^. Three-dimensional periodic boundary conditions were applied to each system. A 2 fs time step was used, and the nonbonded interaction neighbor list was updated every 20 steps. A 1.2 nm cutoff was used for the electrostatic and van der Waals interactions. The long-range electrostatic interactions were calculated using the particle-mesh Ewald method after a 1.2 nm cutoff. The bonds involving hydrogen atoms were constrained using the linear constraint solver (LINCS) algorithm. Besides 37 °C, the MMC, NNC, MNC, and NMC systems were simulated for 200 ns at 5 and 67 °C. The input structure for additional simulations was obtained from the 37 °C production MD run. Except for the temperature, other simulation parameters remained unchanged. Molecular visualization and images were rendered using PyMol,^70^ VMD,^71^ and YASARA software suites. Data analysis and plotting were performed using in-house Python scripts based on publicly hosted Python packages, such as matplotlib, scipy, and MDAnalysis.^72^

### Principal Component Analysis

The MD simulation trajectories were analyzed using in-house scripts to derive 15 features describing aspects of form, size, and hydration of each construct in the last 200 ns of simulation. These variables include (i) end-to-end distance between the three arms (F1–F3); (ii) the radius of gyration (*R*_g_) of each arm and the branching point (S1–S4); and (iii) the average number of water molecules in the proximity of each domain and the average number of hydrogen bonds between each domain and surrounding water molecules (H1–H8), Table S4. This information is used to generate a labeled data set containing 1920 data points (15 features × 4 constructs × 2 temperatures × 16 snapshots sampled within 170–200 ns with 2 ns intervals) as the input for PCA. First, all measurements were standardized using *z*-scoring (i.e., mean equal 0 and standard deviation of 1) to ensure that differences in the scale and nature of these features do not bias the PCA results. The method of Horn’s parallel analysis was used to select components with eigenvalues greater than principal components for a control data set with identical dimension but generated “randomly” using 1000 Monte Carlo simulations at 95 percentile (Figure S21).^73^ The first three principal components that account for 75% of the observed variations were used for the analysis.

### Statistical Analysis

Statistical analysis including PCA was performed using GraphPad Prism 9.2. The output of PCA analysis (PC and loading scores) was imported into OriginPro 2012b (version 9.8.5.204) for visualization and for calculation of 95% confidence ellipsoids in Figure 5. The error bars for all DLS measurements represent the standard deviation of three measurements. TEM images were analyzed using ImageJ and the size, length, area distribution histograms were prepared in Prism.

## Results

### Molecular Design of Star Amphiphiles

**Figure 1:**
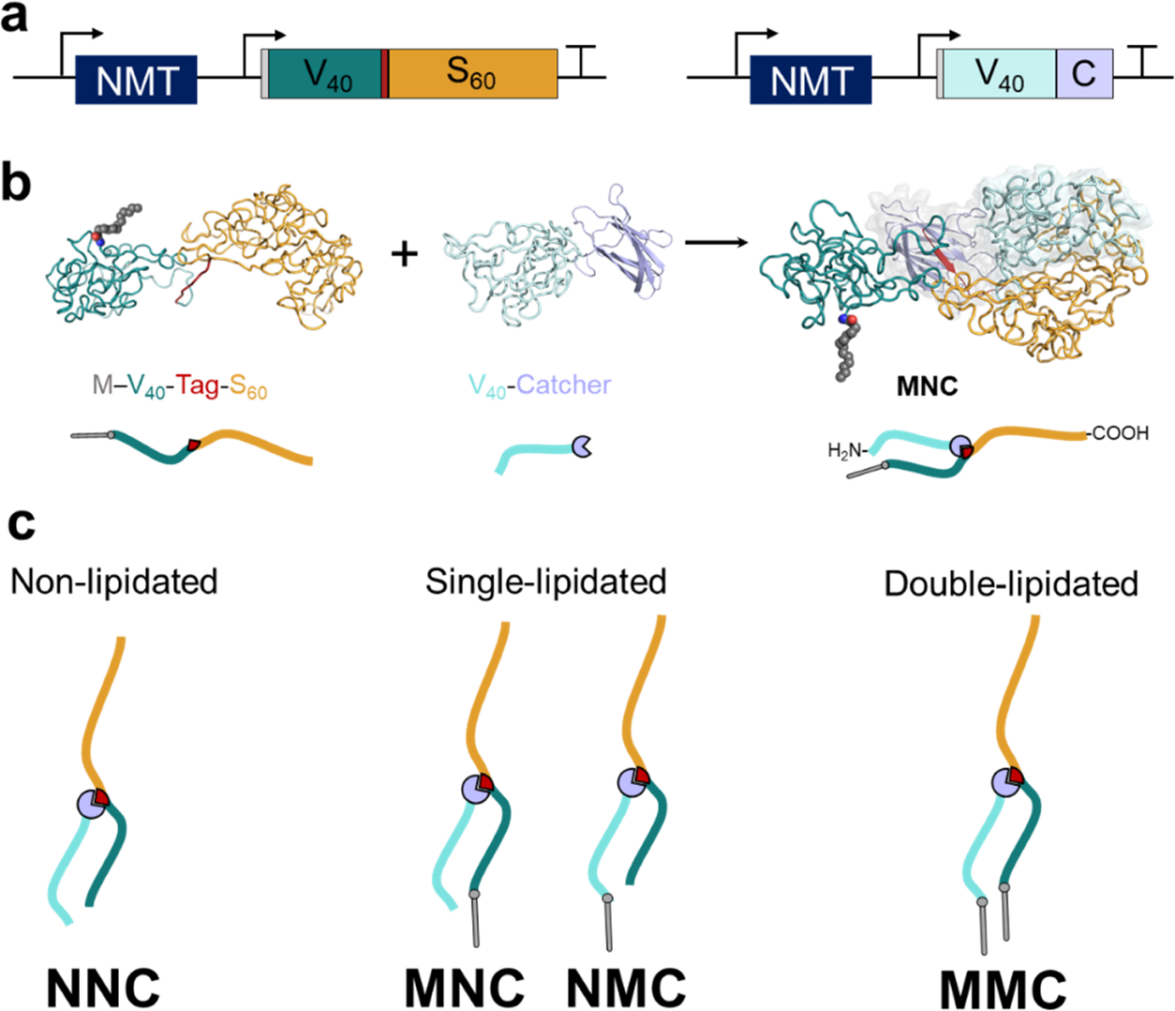
Synthesis and nomenclature of miktoarm star amphiphiles. (a) Architecture of plasmids used for the synthesis of SAFE’s linear building blocks. All genetic elements necessary for the biosynthesis of SAFEs—expression followed by lipidation and branching PTMs—were encoded in orthogonal bicistronic plasmids: (1) ELP arms; (2) N-myristoyltransferase (NMT); and (3) bipartite SpyTag/Catcher proteins. (b) Schematic of the reaction between two model linear building blocks to generate a miktoarm star. (c) Identity of ELP’s guest residue (i.e., hydrophobic valine or hydrophilic serine) and the lipidation pattern of hydrophobic arms define the amphiphilic architecture of each construct. SAFE constructs are labeled using a three-letter code based on the identity of the functional group terminating each arm. “N” and “M” refer to the free amine (unmodified) or myristoyl (modified) hydrophobic arms, and C corresponds to the carboxylic acid of a hydrophilic arm. NNC—nonlipidated, MNC and NMC—single-lipid, and MMC—double-lipid amphiphiles. The first two letters refer to hydrophobic arms that are linearly fused to serine block or catcher domain, respectively.

The miktoarm star amphiphiles studied here have three distinct design elements—ELP arms, branch point, and lipid. This design was informed by our work on mono-lipidated proteins, which formed polydisperse wormlike micelles and fibers after aging or thermal annealing.^74−76^ Thus, we designed a topologically asymmetric building block by selecting two different ELPs as hydrophobic and hydrophilic arms to control the extent of aggregation along the cylinder’s main axis. Hydrophobic arms (A) contained 40 repeats of GVGVP (V_40_), while the hydrophilic arm (B) contained 60 repeats of GSGVP (S_60_). To create the branched topology, we placed the SpyTag at the interface of the hydrophobic and the hydrophilic arms and placed the SpyCatcher at the C-terminus of the second hydrophobic block (i.e., A-Tag-B and A-Catcher). Catcher/Tag pairs post-translationally form an isopeptide bond to create the core of the miktoarm star (A_2_B). Both hydrophobic arms had free N-termini, while the hydrophilic arm contained a carboxylate group. We therefore refer to the nonlipidated constructs as NNC. Since myristoylation (M) occurs at the protein N-termini,^77^ three distinct SAFE constructs are biosynthetically accessible in this topology: one double-lipid (MMC) and two single-lipid (MNC and NMC) amphiphiles, which are distinguished by the location of the lipid. For MNC, “M” is attached to the hydrophobic arm linearly fused to the SpyTag sequence, while in NMC, it is attached to the hydrophobic arm linearly fused to SpyCatcher (Figure 1).

### Recombinant Synthesis, Purification, and Molecular Characterization of SAFEs

To biosynthesize SAFEs, we combined the necessary genetic elements on two bicistronic plasmids (Figure 1a and Table S1): (1) V_40_-Tag-S_60_; (2) V_40_-Catcher; and (3) N-myristoyl-transferase enzyme (NMT), which lipidates the N-glycine of hydrophobic arms when they are fused to a peptide substrate of NMT.^75^ These two plasmids can be used for recombinant expression and lipidation of individual components in separate cells. Combining these lipid-modified building blocks in the second step yields miktoarm stars with the desired lipidation pattern (Figures 1b and S2). This two-pot method provided tight control over the production of constructs with asymmetric lipidated tails and was useful for generating the six linear controls (Figure S3). To reduce the number of synthetic and processing steps, we also demonstrated that it is possible to biosynthesize constructs in one pot by coexpression of NMT, V_40_-Tag-S_60_, and V_40_-Catcher in one cell (Figure S4).

Each construct was purified by leveraging the temperature-triggered phase behavior of ELP arms^78^ and characterized using high-performance liquid chromatography and mass spectrometry to confirm its purity and identity (e.g., the regio-/chemo-selectivity of modification), Figures S5–S7.

**Figure 2:**
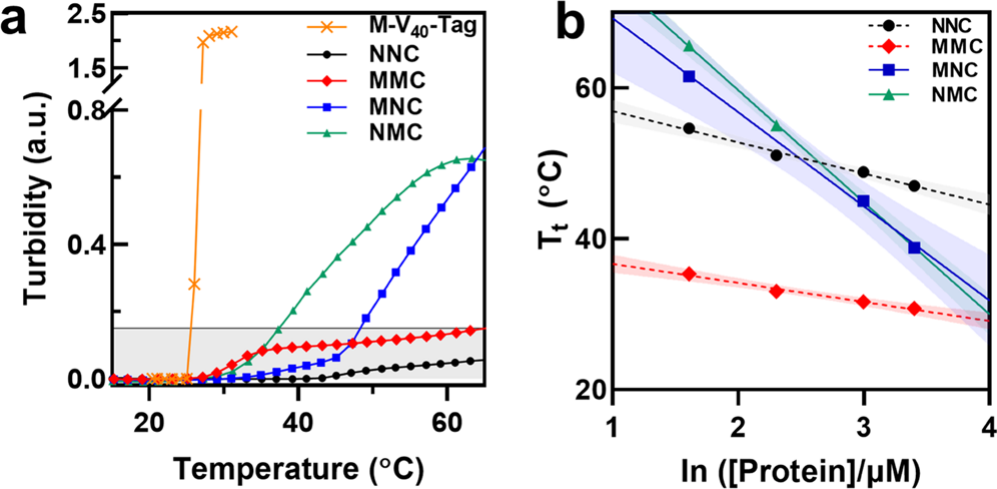
Lipidation patterns modulate the thermoresponse of star amphiphiles. (a) Turbidity profiles of SAFE amphiphiles and M-V_40_-Tag (a linear control) at 20 μM in PBS as a function of temperature. The solution of single-lipid constructs (MNC and NMC) was noticeably more turbid than NNC or MMC (shaded gray area) at *T* > *T*_t_. The thermal behavior of single-lipid amphiphiles showed subtle differences based on the location of the attached lipid. (b) Concentration dependence of SAFE’s transition temperatures. The shaded area represents a 90% confidence interval for the fitted line. The *T*_t_ of NNC/MMC exhibited lower concentration dependence (shallower slope) than single-lipid amphiphiles.

We then used different biophysical and soft-matter characterization methods to test the hypothesis that the lipidation pattern modulates thermoresponse and nanoassembly of SAFEs. To do so, we first characterized the thermal response of SAFE constructs using a temperature-programmed turbidimetry assay (Figure 2) as the external temperatures regulate the solubility and assembly of ELP arms. Canonical ELPs exhibit lower critical solution temperature (LCST) phase transition.^34,79^ At T > LCST, the ELP–ELP interaction is more favorable than ELP–water, resulting in an attractive interaction that can drive the self-assembly of constructs.

### Lipidation Pattern Modulates the Thermoresponse of SAFEs

Figure 2a depicts the turbidity profile for solutions of star amphiphiles (20 μM in phosphate-buffered saline, PBS) and a representative linear control (M-V_40_-Tag) as a function of temperature (15–65 °C), see Figure S10 for turbidity plots of other controls. The turbidity profiles of SAFEs exhibit distinct differences from those of ELPs or lipidated ELPs (e.g., M-V_40_-Tag). Canonical ELPs exhibit a sharp and cooperative LCST behavior that is characterized by a rapid increase in solution turbidity over a narrow temperature range. Consequently, their transition temperature (T_t_) is often defined as the inflection point of the turbidity–temperature plots. Although this “inflection point” method offers advantages such as less dependence on experimental variations in heating rate or protein concentration, it fails to capture (quantify) the cooperative nature of the protein phase separation. As discussed below, the turbidity profiles of star amphiphiles were sigmoidal, but depending on the lipidation pattern, each construct had a different: (1) cloud point temperature (*T*_cp_), the temperature at which turbidity starts to increase; (2) maximum solution turbidity (AU_max_) at 65 °C; and (3) curve steepness (i.e., the rate of turbidity increases as a function of solution temperature).

The *T*_cp_ was inversely correlated with the number of lipids attached to SAFEs: MMC ∼25 °C < NMC and MNC ∼30 °C < NNC ∼45 °C. Intriguingly, both non- and double-lipidated constructs (NNC and MMC) were noticeably more transparent at elevated temperatures (AU_max_ < 0.15) in comparison to single-lipid amphiphiles (MNC and NMC, AU_max_ > 0.6), Figure 2a, shaded area. We inferred that this difference in turbidity indicates that single-lipid constructs undergo liquid–liquid phase separation at elevated temperatures and form mesoscale coacervates that strongly scatter visible light, similar to the behavior of ELPs or their lipidated analogues. On the other hand, the low turbidity of NNC and MMC is consistent with the formation of smaller nanoscale assemblies, as seen in linear ELP block copolymers above the LCST of the hydrophobic block.^80−82^

To quantify differences in steepness, we fitted the turbidity profile to a variation of the Hill saturation function and determined the Hill slope as a measure of cooperativity in the phase behavior for each construct. For example, the Hill slope for lipidated M-V_40_-Tag is *h* > 100, which is consistent with the behavior of canonical ELPs and their lipidated analogues. For comparison, the Hill coefficient for NNC and MMC was ∼20, while the Hill slope was significantly smaller for MNC or NMC (∼7–8), *F* (3,10) = 30.21, *p* < 0.0001. We interpreted these differences as the reduction in the cooperativity of two hydrophobic arms when their lipidation pattern is different. This could occur if myristoylation resulted in the assembly of constructs in such a way that the two arms occupied different locations in the assembled structure. Intriguingly, the behavior of single-lipid amphiphiles was also noticeably different from each other, despite having similar Hill coefficients. Together, these differences indicate that the lipidation pattern modulates each construct’s size and the kinetics of phase separation, as turbidity is caused by the scattering of incident light by SAFE assemblies as the temperature is increased.

Figure 2b shows that the lipidation pattern also modulates the concentration dependence of *T*_t_ in SAFE constructs in the studied range (5–30 μM, Figure S8). Notably, the *T*_t_ for MMC and NNC exhibits a lower concentration dependence than MNC and NMC (i.e., the slope of lines for NNC and MMC are −4.13 and −2.53 °C compared to −12.47 and −14.85 °C for MNC and NMC, respectively). Similarly, the *T*_t_ of linear nonlipidated (Figure S10) controls exhibited a steep concentration dependence, while the LCST of myristoylated controls was less dependent on concentration. This observation indicates that the lipidation pattern modulates the inter-/intramolecular nature of protein interactions that drive phase separation. Although quantitative models have been developed to predict the LCST of linear ELPs and their block copolymers as a function of molecular features and solution conditions (i.e., the polarity of the guest residue, ELP length, concentration, ionic strength, etc.), the influence of nonproteinogenic motifs (lipid) or nonlinear topologies (branched, dendritic, etc.) is less understood.^83−87^ Additional work is needed to elucidate these principles in noncanonical systems.

Results of turbidity experiments revealed two insights: (1) the thermal behavior of the symmetric constructs NNC and MMC was noticeably different from the asymmetrically lipidated constructs MNC and NMC. (2) Constructs that were identical except for lipid location (i.e., MNC vs NMC) have divergent thermoresponses. We hypothesized that these observed differences originate from the temperature-dependent assembly of SAFEs to different nano-/mesoscale structures.

### Lipidation Patterns Modulate the Nano- and Mesoscale Assembly of SAFEs

**Figure 3:**
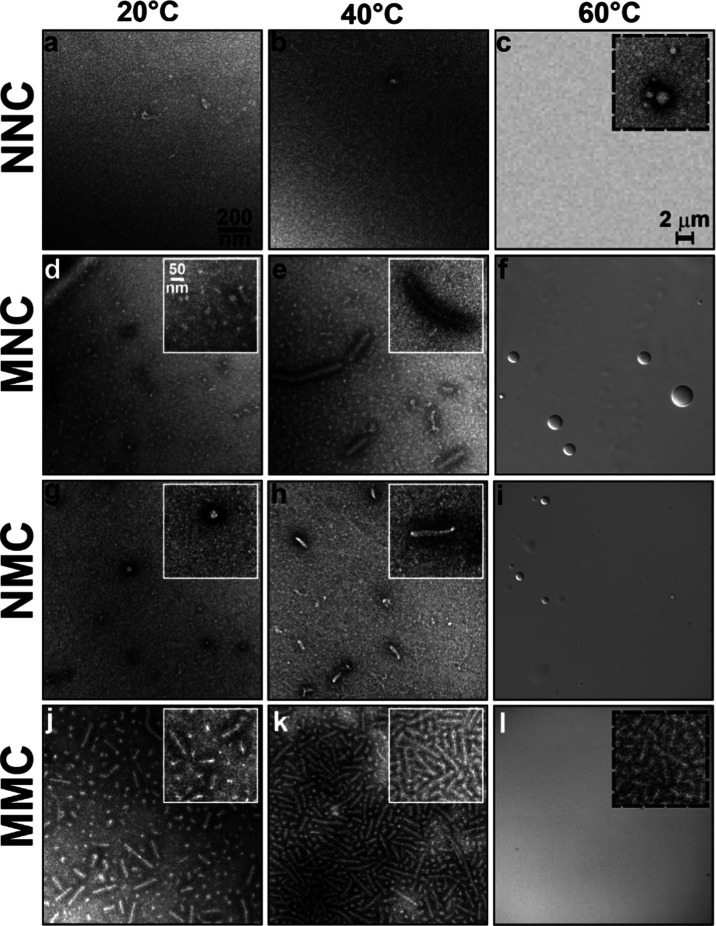
Microscopic characterization of the lipidated SAFE’s assembly at nano-/mesoscale. (a–c) NNC; (d–f) MNC; (g–i) NMC; and (j–l) MMC. NNC remains as unimer and no assembly is observed at 20 and 40 °C. At 60 °C, NNC formed spherical micelles (inset in (c), 16 ± 4 nm) and did not show any bulk-phase separation in DIC. MNC forms a mixture of spherical and elongated aggregates at 20 °C and high-aspect-ratio bottle brushes with a well-defined diameter (75 ± 20 nm) but polydisperse lengths (261 ± 172 nm) at 40 °C. NMC forms spherical assemblies at 20 °C and nanotapes at 40 °C. Compared to MNC bottle brushes, the core of these structures (visualized as white areas) was wider, but their corona was less resolved. In contrast, MMC formed a mixture of spherical and elongated nanoworms at 20 °C. The spherical assemblies were converted to nanoworms at 40 °C with a well-defined size. At 60 °C, both single-lipid constructs undergo liquid–liquid phase separation and form micron-size coacervates (consistent with turbidimetry and DLS data). However, MMC nanoworm aggregates were stable at high temperatures (inset in (l)), and no bulk-phase separation was detected in DIC.

To test this hypothesis, we used microscopy to visualize the assembly of SAFEs at three different temperatures (20, 40, and 60 °C), Figure 3 and Table S11. Transmission electron microscopy (TEM) was used to characterize nanoscale assemblies. NNC only formed small spherical assemblies at elevated temperatures (16 ± 4 nm, Figures 3c and S11). All lipidated constructs formed temperature-responsive nanoassemblies. MNC formed a mixture of isotropic spherical aggregates and ill-defined high-aspect-ratio structures at 20 °C (Figure 3d). Increasing the temperature to 40 °C resulted in supramolecular bottle–brush assemblies with a narrow core (white area) and a dense brush layer (darker area), as shown in Figure 3e. In contrast, NMC predominantly formed spherical assemblies at 20 °C, which transitioned into a different type of wormlike micelles (nanotape) at 40 °C (Figure 3g,h). The cores in these tapes were noticeably larger than those of bottle brushes, while their corona was less visible when compared to the brushlike structures. Figure S12 shows the TEM images of single-lipidated constructs at a higher magnification.

Meanwhile, MMC (Figure 3j,k) first assembled into a mixture of spherical particles and nanoworms at 20 °C. As the temperature increased to 40 °C, the number of spherical aggregates decreased and a more homogenous mixture of slightly longer nanoworms were formed. At 60 °C, these nanoworms dominated the observed nanostructure, but no statistically significant difference between nanoworms length distributions is detected at 40 or 60 °C using unpaired, two-tailed *t*-test, *t* (348) = 0.58, *p* = 0.56.

We also used cryo-TEM to image assemblies in their native hydrated states at 20 and 40 °C and confirmed the findings from negatively stained images (Figure S12): (1) both NMC and MNC form spherical assemblies at low temperatures, which are then converted to rodlike assemblies at higher temperatures; (2) MMC forms nanoworms with low polydispersity at 40 °C.

Differential interference contrast (DIC) microscopy confirmed the effect of lipidation patterns on the mesoscale assembly of SAFE constructs. Both single-lipid amphiphiles underwent liquid–liquid phase separation and formed micron-size coacervates at 60 °C (Figure 3f,i). In contrast, NNC and MMC did not undergo bulk-phase separation from the solution, and no coacervates were observed (Figure 3c,l). Consistent with the observed thermal stability, circular dichroism also confirmed that the secondary structure of NNC or MMC does not change when proteins are heated up to 65 °C (Figure S13).

**Figure 4:**
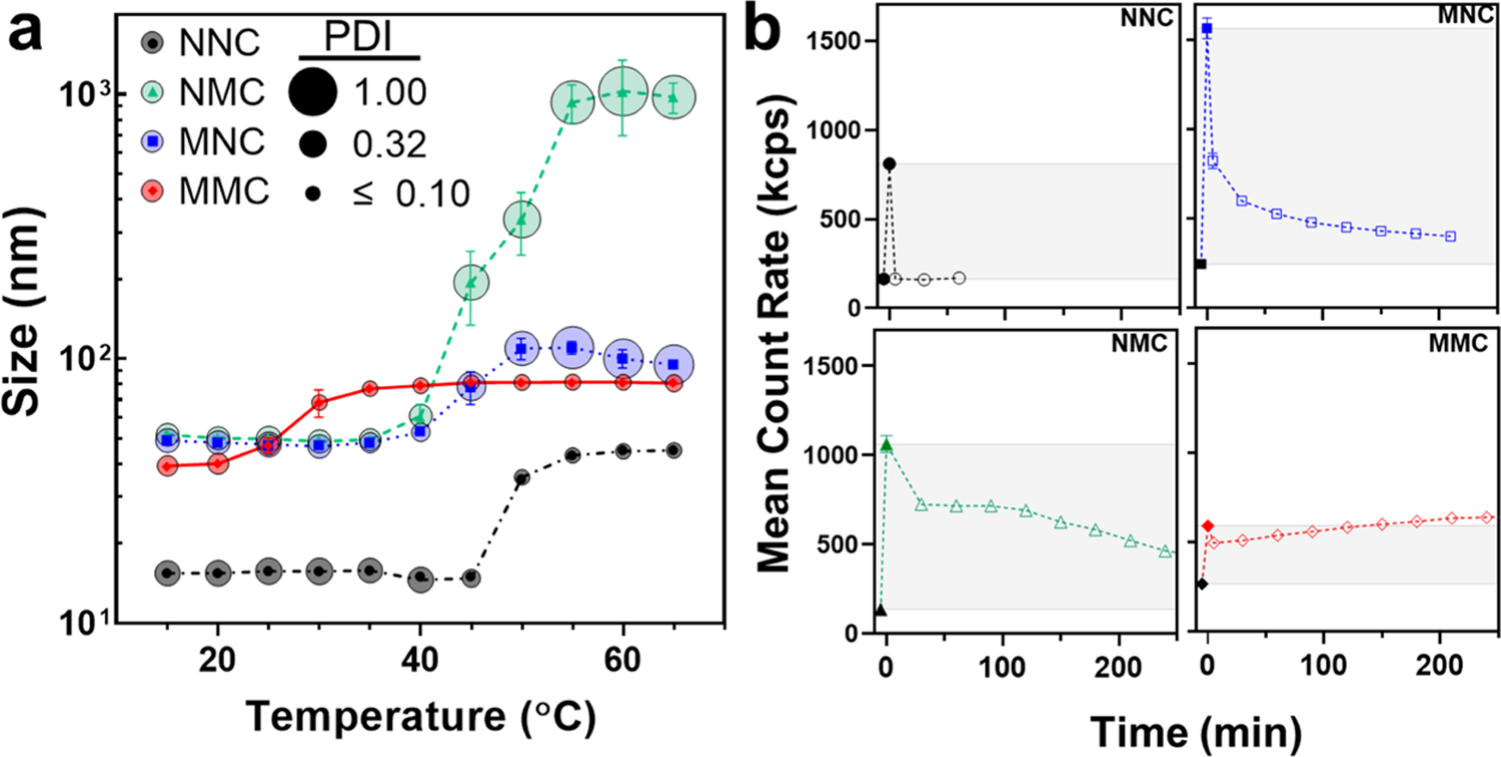
Dynamic light scattering confirms that the lipidation pattern modulates the temperature-dependent (dis)assembly of star amphiphiles. (a) Bubble plot summarizing the average hydrodynamic diameter (*Z*_avg_, symbols) and the polydispersity index (PDI, the area of the bubble) of SAFEs as a function of temperature. NNC only formed small assemblies when heated above 50 °C, while all lipidated samples assembled even below their *T*_t_. The size of lipidated SAFE assemblies increased with temperature, albeit a divergent behavior was observed depending on their lipidation patterns. The size and PDI of NMC and MNC increased above *T*_t_ because they formed a mixture of coacervates with different sizes above 40 °C (Figures S11 and S12). The size of MMC assemblies initially increased with temperature but remained unchanged above 30 °C with a low PDI (<0.1). (b) DLS is used to monitor the kinetics of the disassembly of star amphiphiles. In each panel, scattering intensity at 20 and 50 °C are represented with filled black and colored symbols. After cooling the sample to 20 °C, the scattering intensity is monitored over time (open symbols). NNC showed complete reversibility immediately after cooling. The scattering intensity of both MNC and NMC decreased with time upon cooling, albeit their disassembly kinetics were different. MMC remained stable at 20 °C, with even a modest increase in the scattering profile. Incubation at 4 °C was required to disassemble the MMC aggregates (data not shown). Error bars are standard deviations of three measurements. Lines connecting the data points are added as a visual reference.

To complement the results of microscopy, we used dynamic light scattering (DLS) to investigate the assembly of SAFE in PBS as a function of temperature (15–65 °C at 5 °C increments). The cumulant method was used to analyze DLS autocorrelation functions (Figure S15) and to derive the size and dispersity (*Z*_avg_ and polydispersity index, PDI) of SAFE assemblies at various temperatures (Table S2). Figure 4a shows the results of this analysis as a bubble plot with the center of each circle representing *Z*_avg_ and the area of each circle representing PDI. *Z*_avg_ is the intensity-weighted mean hydrodynamic size of the ensemble collection of particles, and PDI represents the dispersity of this ensemble, 0 (monodisperse) < PDI < 1 (polydisperse).

As shown in Figure 4, the size of unmodified stars (*Z*_avg_ = 12 nm, black lines and circles) at low temperatures (<45 °C) suggests a lack of assembly in this range. Above 50 °C, *Z*_avg_ increased to ∼30 nm, indicating the formation of small assemblies at higher temperatures. The low PDI of these samples suggests that they are spherical, consistent with the formation of spherical micelles in TEM studies. The single-lipid NMC and MNC formed assemblies of similar sizes and PDI at low temperatures (blue and green lines and bubbles). As *T* > *T*_cp_, both samples started to form larger aggregates, but their behavior started to diverge. The *Z*_avg_ for NMC exceeded 1 μm, while the *Z*_avg_ of MNC was significantly smaller (∼100 nm). This is consistent with the formation of coacervates for NMC, though it reflects the unequal contribution of small and large MNC particles.

Meanwhile, the behavior of MMC was distinctly different. At low temperatures, MMC assembled into aggregates with an average size of 30 nm and a lower PDI compared to NMC and MNC. As *T* > *T*_cp_, the aggregate size started to increase and reached ∼80 nm at 30 °C. Increasing the temperature to 65 °C did not result in a significant increase in aggregate size. Notably, the PDI of single-lipid amphiphiles increased with temperature (approaching the maximum theoretical value of 1), while the PDI of MMC decreased with temperature. This is consistent with the formation of a more homogeneous nanoworm assembly population for MMC, which drastically contrasts with the formation of polydisperse coacervates observed for NMC and MNC at higher *T* > *T*_t_.

Finally, we used both turbidimetry and DLS to investigate the reversibility of the phase transition and nanoassembly of star amphiphiles. NNC exhibited a reversible LCST behavior, characterized by the reduction in the solution turbidity to its initial state and the dissolution of NNC particles into unimeric chains following cooling (Figure S18). However, lipidated samples exhibited different degrees of reversibility in their phase behavior—NMC (72%), MNC (34%), and MMC (18%). Similarly, we measured the average scattering intensity (DLS mean count rate) for each sample at 20, 50 °C, and subsequently at 20 °C over time. The larger assemblies scatter light more intensely, so the reduction of scattering profile as a function of time correlates with the disassembly of each construct (Figure 4b). Consistent with the turbidimetry, DLS showed that larger structures formed by lipidated proteins remain stable even after cooling the solution below LCST. These results suggest that while the coacervates of NMC and MNC dissolve rapidly below their LCST temperatures, the nanoassembled structures of NMC, MNC, and MMC persist in solution. Prolonged incubation (>5 h) of samples below *T*_cp_ is necessary for the scattering profile to return to its original values (Figure 4b), which suggests that lipidation and self-assembly slow the kinetics of ELP hydration (dissolution) at the molecular level. Dual lipidation of MMC can also reduce the kinetics of (ELP) chain exchange, similar to the behavior of ABA triblock copolymers or polymers modified with hydrophobic groups at both ends.^38^

Results of turbidimetry, scattering, and microscopy experiments consistently demonstrate the following points: (1) lipidation pattern changes the assembly and thermoresponse of SAFEs. (2) The changes in material properties as a function of temperature for the non- and double-lipidated constructs (NNC and MMC) differ considerably from the behavior of single-lipid SAFEs (MNC and NMC). (3) Intriguingly, differences in the lipidation site resulted in subtle differences in the assembly and thermoresponse of single-lipid amphiphiles (Figure S19).

These findings confirm our hypothesis that the material properties of SAFE can be modulated by changing their lipidation patterns and amphiphilic architecture. However, they also hint a complex interplay between lipidation pattern, structure, and energetics of chemically and topologically modified SAFEs. These observations motivated our use of MD simulations to gain molecular-level insight into the interplay between the physicochemistry of lipids and the composition of the various constructs.^88,89^ To compute in silico properties, we focused on unimer dynamics that are precise yet have relatively low computational cost while being mindful that thermoresponse and assembly are bulk properties (i.e., impacted by interactions between multiple chains). However, past studies have shown that single-chain properties such as hydration can reliably predict LCST behavior for linear ELPs.^90,91^ Similarly, we suggest that the physicochemical interplay between and among protein, lipid, and branching point modulates the key drivers of bulk properties at the single-chain level. The MD simulations were used to compute a series of structural and physicochemical properties corresponding to the size, shape, and hydration of constructs at 5, 37, and 67 °C, corresponding to temperatures below, around, and above LCST of all constructs.

### Intramolecular Structure of SAFE Unimer Is Affected by the Lipidation Pattern

**Figure 5:**
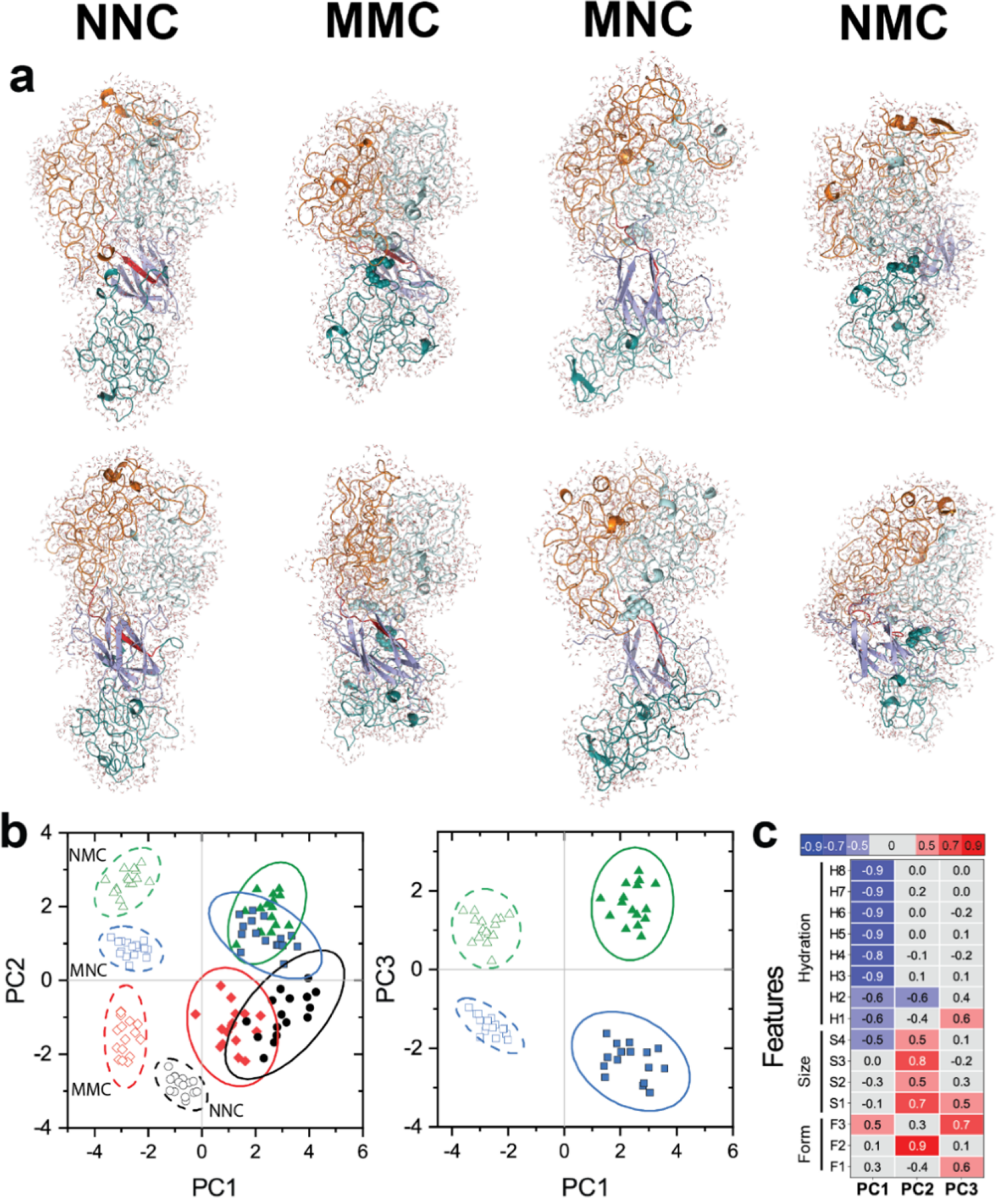
Lipidation pattern alters the physicochemical properties of star amphiphiles at the single-chain level. (a) Atomistic conformations of NNC, MMC, MNC, and NMC structures (front and back, cartoon representation) along with their first hydration shell (dots) at 37 °C. Color scheme for the structures: SpyCatcher (light blue), SpyTag (red), V_40_ fused to SpyCatcher (cyan), V_40_ fused to SpyTag (teal), and S_60_ (orange). The attached lipids are shown as spheres and colored based on the color of the attached ELP. (b) Principal component analysis sorts MD simulation results into largely nonoverlapping clusters. PC axis 1 correlates with temperature changes, while PC2 discriminates single-lipid amphiphiles from symmetrically non- or double-lipidated NNC and MMC. PC3 captures variations between the single-lipid constructs MNC and NMC. In both panels, the open symbols and dashed lines refer to simulations at 5 °C, while filled symbols refer to results at 67 °C. NNC (circle), MMC (diamond), MNC (square), and NMC (triangle). (c) Heat map depicts the contribution (loading) of each molecular feature to PC1–PC3, with blue and red representing negative or positive loadings. The lipidation pattern and temperature modulate size, shape (form), and hydration of star amphiphiles. F1–F3 represent the pairwise distance between different arms; S1–S4 represent the size of each arm and the branching point. H1–H8 represent the number of water molecules in the hydration shell and the number of hydrogen bonds between the solvent and each domain. See the Methods section for the definition of each variable.

The trajectories obtained in the last 200 ns of MD simulations were used to derive 15 parameters related to different aspects of amphiphilic architecture (size, form(shape), and hydration) from the trajectories at 100 ps intervals (Figure S20). These parameters include (a) radius of gyration (*R*_g_) of each arm and the branching point; (b) end-to-end distance between the three arms; (c) the number of water molecules in the first hydration shell of the molecule (3.2 Å cutoff); and (d) hydrogen bonds between the protein and water (Table S4). The equilibrium structures of NNC, MNC, NMC, and MMC show how single-tail and double-tail modifications alter the intramolecular structure of constructs (Figure 5a). We then used principal component analysis (PCA),^92^ an unsupervised machine learning (ML) algorithm, for clustering simulation output parameters in a space defined by the first three principal components (PCs), which accounted for at least 75% of the variation in the original data set. As shown in Figure 5b, constructs with different amphiphilic architectures were separated into nonoverlapping areas of space defined by these PCs. Specifically, PC1 was strongly correlated with the effect of temperature, as the clusters for all constructs shift to the right as the temperature is increased. Moreover, PC1 could discriminate between nonlipidated and lipidated constructs. PC2 captured differences between single-lipid constructs and non- or double-lipidated constructs, MNC/NMC vs NNC/MMC. PC3 discriminated lipidated constructs as well as single-lipid constructs (MNC vs NMC). The separation between these clusters, which is consistent with experimental findings, strongly supports the notion that single-chain simulations can capture the effect of lipids and temperature on the structure, hydration, and energetics of SAFE constructs. Moreover, these results demonstrate that the combination of MD simulations and ML algorithms can detect subtle differences in the behavior of highly homologous amphiphiles, which should facilitate the design of soft materials.

Because PCs include the varied influences of original features,^93^ differences between the clusters can be traced back to changes in these features as a function of amphiphilic architecture or temperature. This information is captured in loading plots (Figure 5c), which elucidates the contribution of features to each PC on a normalized scale, −1 to 1. For instance, features corresponding to hydration are negatively correlated with PC1. As the temperature is increased (along the PC1 positive axis), constructs are dehydrated. This correlation is intuitive given the LCST behavior of ELP and is consistent with previous computational studies.^94^ A detailed analysis of loading plots also revealed the subtle biophysical interplay between the different components of the molecular syntax. For example, dehydration of the hydrophobic arm fused to SpyCatcher showed a weaker correlation with temperature (cf. loading of H1 and H2 with H3–H8 in PC1, −0.6 vs −0.9), but dehydration uniquely contributed to PC2 and PC3, which discriminated between SAFEs with different lipidation patterns.

**Figure 6:**
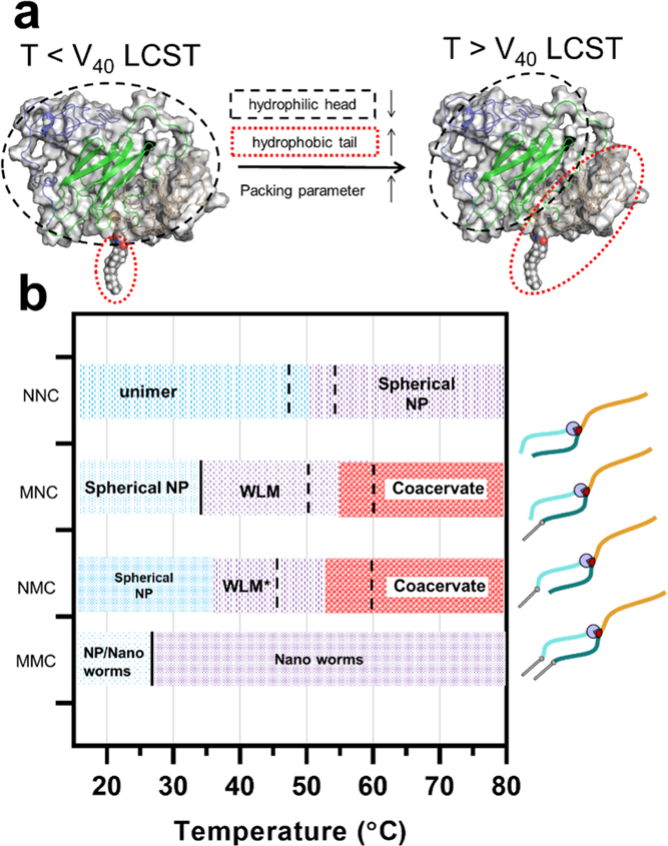
(a) Increasing the temperature above the LCST of hydrophobic domains increases the packing parameter of SAFEs, which triggers the structural transition from spherical to rodlike assemblies. (b) Schematic phase diagram for the temperature-dependent assembly of star amphiphiles derived from the collection of turbidimetry, DLS, and TEM studies. The dashed vertical lines show the approximate temperature range for concentration-dependent transitions in nano- or mesoassemblies. The solid vertical lines are added to denote concentration-independent transitions. The combination of dual lipidation and branching in MMC is necessary to form nanoworms over a broad window of temperature and concentration ranges. WLM is wormlike micelles. Figure S19 shows the phase diagram of controls.

Extending this analysis to other features enables us to parse the contribution of lipidation patterns to the observed differences between the constructs and identify similar intuitive and subtle variations in size, shape, or hydration of each construct with high resolution. An analogy to the “packing parameter,” which predicts the assembly of amphiphiles based on geometric considerations such as size and shape of hydrophobic/hydrophilic moieties, is illustrative.^95^ Below LCST, all ELP domains (two V_40_ and S_60_) are completely hydrated, and the SAFE’s assembly is driven by the aggregation of the lipid tail. The mismatch between the size of hydrophobic and hydrophilic domains results in a small packing parameter, which is a predictor of assembly into spherical micelles (consistent with DLS and TEM experiments).^96^ As the temperature increases above the LCST of the hydrophobic domains (i.e., V_40_), these domains will dehydrate and become hydrophobic. This transition alters the balance of hydrophobic/hydrophilic interactions and effectively increases the packing parameter (as hydrophobic domains are enlarged and hydrophilic domains are shrunken), Figure 6a.

## Discussion

Controlling the length of 1D cylindrical assemblies—a prerequisite for the formation of stable nanoworms—requires balancing the delicate interactions of building blocks along the main axis versus the endcap region.^97^ For most amphiphiles, the addition of a monomer to the cylinder length is a noncooperative process.^98,99^ That is, the free energy of micelle growth does not change as the aggregation number is increased. This property hinders the thermodynamic control over the growth process and promotes the formation of a polydisperse mixture with lengths ranging from nano-to-micrometer. These difficulties may explain why there are few systematic investigations on the preparation of protein nanoworms in the literature.

We hypothesized that changing the linear topology of protein fusions may broaden the stability of nanoworms in the phase diagram. To test this hypothesis, we combined two types of PTMs, lipidation and branching, to synthesize high-molecular-weight, sequence-defined star amphiphiles with unique and programmable amphiphilic architecture defined by the composition of proteins and lipidation patterns. In the absence of lipidation, NNC only self-assembles into spherical micelles—highlighting that the topology factor alone is insufficient to induce the assembly of nanoworms. This is consistent with the previous work that showed ELP block copolymers assemble into weak micelles as A and B blocks do not strongly repel each other.^100^ On the other hand, while lipidated SAFEs (and the linear controls) can assemble into wormlike micelles, only MMC forms stable nanoworms, while others aggregate into the polydisperse mixture of worms, fibers, or coacervates (Figure 6b). This confirms the role of topological modification in increasing the stability boundaries of nanoworms and highlights the synergy between lipidation and branching post-translational modifications in this system.

We also demonstrated that the lipidation pattern modulates the phase behavior of star amphiphiles. Intriguingly, the addition of a single lipid reduced LCST phase boundaries and promoted macroscopic phase separation above 40 °C. In contrast and counterintuitively, double-lipidated constructs did not show macroscopic phase separation even when heated to 65 °C. While LCST phase behavior is a useful feature for scalable purification of proteins, it also presents an upper operating condition for using these constructs as nanomaterials, since above the cloud point, mesoscale coacervates are formed (Figure S19).^101^ This limits the use of lipid-modified elastin for high-temperature applications such as templated synthesis of nanomaterials. Therefore, our results for MMC may provide a translatable design principle for maintaining the solubility of lipidated constructs even at very high temperatures by avoiding the LCST transition into micron-sized aggregates.

Similarly, DLS and TEM confirmed that the lipidation pattern significantly influences the nanoscale assembly of star amphiphiles as a function of temperature. Importantly, our study shows that a judicious choice of amphiphilic architecture can be used to prepare *adaptive* nanoworms that undergo a shape transformation in response to temperature stimuli. This morphological change, combined with the modulation of phase separation behavior discussed above, increases nanoworms stability even at extremely high temperatures. These characteristics are not found in the protein nanoworm literature; nanoassembly either did not change with temperature or formed large aggregates at elevated temperatures.

To understand the origin of these divergent behaviors, we combined MD simulations of a unimer with PCA to parse the effect of lipidation patterns on the energetics and structure of these hybrid amphiphiles. This integrated approach revealed that lipidation alters the properties (e.g., hydration, size, and conformation) of V_40_ domains, even when the lipid is attached at a distant location. That is, lipidation also enhances compositional differences between A and B blocks, despite the very small size of the lipid. Moreover, in single-lipidated constructs (MNC or NMC), the two V_40_ domains are not equivalent because lipidation alters the hydration pattern, as shown in molecular dynamic simulation and principal component analysis. These variations can explain the observed differences in the nanoscale assembly of these amphiphiles as the function of molecular syntax or temperature. MD simulations are increasingly utilized to provide molecular-level insights that are experimentally unattainable and to explain dynamical behavior observed in self-assembled nanostructures. Our results highlight the power of MD simulations to account for the effects of complex sequence-encoded interactions.

## Conclusions

This study presents several notable outcomes: First, it provides a straightforward road map to synthesize adaptive, recombinant nanoworms. Due to their amphiphilicity, these nanoworms can easily solubilize hydrophobic chemotherapeutics without resorting to complex, inefficient, and time-consuming conjugation/purification protocols. The recombinant nature of this system enables the fusion of genetically encoded bioactive or targeting peptides, which can be used to optimize the delivery and efficacy of these nanoplatforms.

Second, it significantly expands the design space of hybrid protein-based-materials by demonstrating the compatibility between two classes of PTMs, lipidation and protein branching. We anticipate that these methods will be generalizable to other classes of proteins and PTMs. Thus, this work will advance the study and design other hybrid systems, such as lipidated resilin with upper critical solubility phase behavior,^102^ or proteins modified with other classes of lipids (e.g., cholesterol^103^) or charged PTMs such as phosphorylation.^104−106^

Third, integration of experiment, simulation, and data analytics provides a road map to move the synthesis of hybrid functional biomaterials beyond current ad hoc approaches into the realm of predictive design. Traditional brute-force material design, synthesis, and characterization strategies to elucidate the design principles of these hybrid materials are impractical given the large design space resulting from the orthogonality of protein, lipidation, and branching “building blocks.” Our proposed alternative strategy is to use MD simulations and data analytics to survey quickly and less expensively the hybrid design space and then experimentally verify results. While commonly used in biophysical and biochemical studies,^107^ MD simulations is an emergent tool to design soft materials.^108−111^ However, realizing the full potential of this method requires new approaches to reduce the computational cost of multiscale modeling required to predict the properties of desired materials.^22,112−115^ As shown here, the integration of machine learning can provide insights into design principles—a thermodynamically grounded understanding of the contribution of molecular syntax to a programmable assembly of hybrid materials. Elucidating these principles will foster the development of next-generation biomaterials and therapeutics whose forms and functions rival the exquisite hierarchy and capabilities of biological systems.

## Supporting Information Available

Sequence of proteins, experimental details of cloning, expression, modification, purification, and characterization, and supplementary tables and figures (PDF)

## Abbreviations

DIC: differential interference contrast
DLS: dynamic light scattering
ELP: elastinlike polypeptides
LCST: lower critical solubility temperature
PDI: polydispersity index
PBS: phosphate-buffered saline
PCA: principal component analysis
SAFE: star-shaped amphiphilic fatty acid-modified elastinlike polypeptides
