# Supplementary material for: Adaptive Recombinant Nanoworms from Genetically Encodable Star Amphiphiles
Source: Biomacromolecules. 2021 Dec 23;23(3):863–76. doi: 10.1021/acs.biomac.1c01314 (PMC8924867; doi:10.1021/acs.biomac.1c01314)
Supplement: Supplementary file 1 — bm1c01314_si_001.pdf [file bm1c01314_si_001.pdf]

Adaptive Recombinant Nanoworms from Genetically Encodable Star Amphiphiles

Md Shahadat Hossain,[1] Jingjing Ji,[2] Christopher Lynch,[1] Miguel Guzman,[1] Shikha Nangia,[2,3*] and Davoud Mozhdehi[1,3*]

1. Department of Chemistry, Syracuse University, Syracuse, New York 13244, United States; 2. Department of Biomedical and Chemical Engineering, Syracuse University, Syracuse, New York 13244, United States; 3. BioInspired Syracuse: Institute for Material and Living Systems, Syracuse University, Syracuse, New York 13244, USA

Supplementary Information

Table of Contents

1. Materials 1
2. Cloning, DNA, and Proteins' Sequences 2
3. Protein Expression and Purification 3
4. Supplementary Tables 4
5. Supplementary Figures 7
6. References 27

1. Materials

Restriction enzymes, ligase, corresponding buffers, DNA extraction and purification kits, and chemically competent Eb5alpha and BL21(DE3) cells were purchased from New England Biolabs (Ipswich, MA). DNA oligonucleotides and gene fragments were synthesized by Integrated DNA Technologies (Coralville, Iowa). Apomyoglobin, adrenocorticotropic hormone (ACTH), sinapinic acid, alpha-cyano-4-hydroxycinnamic acid, ammonium bicarbonate, and trifluoroacetic acid (TFA) were purchased from Sigma-Aldrich (St. Louis, MO). High-performance liquid chromatography-(HPLC) grade acetonitrile, isopropyl β-D-1-thiogalactopyranoside (IPTG), SnakeSkin™ dialysis tubing with 7 K nominal molecular weight cut off (MWCO), mass spectroscopy grade Pierce™ trypsin protease, tryptone, yeast extract, agar, sodium chloride, ampicillin, phosphate buffer saline (PBS), myristic acid, urea, and ethanol were purchased from

Thermo Fisher Scientific (Rockford, IL). Mini-PROTEAN® TGX Stain-Free™ Precast Gels, Precision Plus Protein™ All Blue Pre-stained Protein Standard, and Precision Plus Protein™ Unstained Protein Standards were purchased from Bio-Rad Laboratories, Inc. (Hercules, CA). The carbon-coated grid (CF300-Cu) was purchased from Electron Microscopy Sciences (Hatfield, PA). Deionized water was obtained from a Milli-Q® system (Millipore SAS, France). Simply Blue™ SafeStain was purchased from Novex (Carlsbad, CA). All chemicals were used as received without further purification.

2. Cloning, DNA, and Proteins' Sequences

The genes encoding SpyTag and SpyCatcher sequences were first cloned into modified pET24a(+) using restriction digest and NEBuilder® HiFi DNA Assembly. The modified pET24a(+) vector, originally developed by McDaniel et al.,[1] contains unique recognition sequences for type IIs restriction enzymes BseRI and AcuI that flank the gene of interest. This feature enables the directional and modular assembly of repetitive protein polymers, i.e., $(GVGVP)_{40}$ and $(GSGVP)_{60}$, and the Spy pairs. In parallel, we first fused the $(GVGVP)_{40}$ gene to the N-termini of SpyTag and SpyCatcher genes. The $(GVGVP)_{40}$-Tag was subsequently fused to the N-terminus of $(GSGVP)_{60}$ in the second round of directional ligation to generate plasmids encoding for linear blocks. To generate myristoylated constructs, these genes were than subcloned into a modified pETDuet-1,[2] a bicistronic vector containing all the necessary genetic elements for N-myristoylation. These elements include the NMT enzyme from *S. cerevisiae* and the site of N-myristoylation: a peptide substrate derived from ARF2 protein. The linear blocks were subcloned downstream of ARF2 recognition sequence (underlined). The schematic of this process is shown in Figure S1. Control plasmids lacking NMT and RS were used to express nonmyristoylated proteins (**Table S1**).

V_{40}-Tag-S_{60}

GLYASKLFSNLGVGVPGVGVPGVGVPGVGVPGVGVPGVGVPGVGVPGVGVPGVGVPGVGVPGVGVPGVGVPGV
GVPGVGVPGVGVPGVGVPGVGVPGVGVPGVGVPGVGVPGVGVPGVGVPGVGVPGVGVPGVGVPGVGVPGVGVP
GVGVPGVGVPGVGVPGVGVPGVGVPGVGVPGVGVPGVGVPGVGVPGVGVPGVGVPGVGVPGVGVPGAHIVMVD
AYKPTKGSGVPGSGVPGSGVPGSGVPGSGVPGSGVPGSGVPGSGVPGSGVPGSGVPGSGVPGSGVPGSGVPGS
GVPGSGVPGSGVPGSGVPGSGVPGSGVPGSGVPGSGVPGSGVPGSGVPGSGVPGSGVPGSGVPGSGVPGSGVP
GSGVPGSGVPGSGVPGSGVPGSGVPGSGVPGSGVPGSGVPGSGVPGSGVPGSGVPGSGVPGSGVPGSGVPGS
GVPGSGVPGSGVPGSGVPGSGVPGSGVPGSGVPGSGVPGSGVPGSGVPGSGVPGSGVPGSGVPGSGVPGSGVP
GSGVPGSGVPGSGVPGY

V_{40}-Catcher

GLYASKLFSNLGVGVPGVGVPGVGVPGVGVPGVGVPGVGVPGVGVPGVGVPGVGVPGVGVPGVGVPGVGVPGV
GVPGVGVPGVGVPGVGVPGVGVPGVGVPGVGVPGVGVPGVGVPGVGVPGVGVPGVGVPGVGVPGVGVPGVGVP
GVGVPGVGVPGVGVPGVGVPGVGVPGVGVPGVGVPGVGVPGVGVPGVGVPGVGVPGVGVPGVGVPGVDTLSGL
SSEQGQSGDMTIEEDSATHIKFSKRDEDGKELAGATMELRDSSGKTISTWISDGQVKDFYLYPGKYTFVETAAPDGY
EVATAITFTVNEQGQVTVNGKATKGDAHIG

SpyTag

Forward	5'-	C	GCA	CAC	ATA	GTA	ATG	GTA	GAC	GCC	TAC	AAG	CCG	ACG	AAG	GGC	TAA	TGA	TAA	TGA	TCT	TCA	G		-3'
Reverse	3'-	CCG	CGT	GTG	TAT	CAT	TAC	CAT	CTG	CGG	ATG	TTC	GGC	TGC	TTC	CCG	ATT	ACT	ATT	ACT	AGA	AGT	CCT	AG	-5'
		G	A	H	I	V	M	V	D	A	Y	K	P	T	K	G	*	*	*	*	S	S	G		

SpyCatcher

ATGGGCGTTGATACCTTATCAGGTTTATCAAGTGAGCAAGGTCAGTCCGGTGATATGACAATTGAAGAAGATAGT
GCTACCCATATTAAATTCTCAAAACGTGATGAGGACGGCAAAGAGTTAGCTGGTGCAACTATGGAGTTGCGTGAT
TCATCTGGTAAAACTATTAGTACATGGATTTCAGATGGACAAGTGAAAGATTTCTACCTGTATCCAGGAAAATATA
CATTTGTCGAAACCGCAGCACCAGACGGTTATGAGGTAGCAACTGCTATTACCTTTACAGTTAATGAGCAAGGTC
AGGTTACTGTAAATGGCAAAGCAACTAAAGGTGACGCTCATATTGGCTAATGATAATGA

3. Protein Expression and Purification

The expression and purification of myristoylated proteins were conducted by adapting a previously established protocol.[2, 3] Protein expression and lipidation were conducted in *E.coli* BL21(DE3) strains harboring a bicistronic pETDuet-1 vector encoding for N-myristoyl transferase (NMT, from *S. cerevisiae*) and either V_{40}-Tag-S_{60} or V_{40}-Catcher. A single colony was used to inoculate a starter culture (2x YT, 4 mL supplemented with 100 µg/mL ampicillin). After overnight growth at 37 °C, the starter culture was used to inoculate a 1L 2x YT culture supplemented with 100 µg/mL ampicillin. The larger culture was incubated with shaking 37 °C at 200 rpm and the growth of the bacteria were monitored by measuring OD_{600} every hour. At OD_{600} value of 0.8, temperature was reduced to 28 °C, and the media was supplemented with 100 µM myristic acid (stock solution prepared in DMSO at 10X working concentration). After 15 min, the protein expression was induced by addition of IPTG to the final concentration of 500 µM. After 6 h of expression, the cells were harvested by centrifugation (3745 × g, 15 min, 4°C) and resuspended in PBS (10 mL/cell pellet of 1L). The harvested cells were stored at -80 °C before purification. After lysis by sonication, the lysate was clarified by centrifugation (22,830 × g, 4 °C, 15 min) and the proteins were purified by leveraging their phase-behavior using inverse transition cycling (ITC).[4] Proteins used for self-assembly studies were purified by reversed-phase HPLC (RP-HPLC) to ensure > 95% purity. Organic solvents were removed by dialyzing the protein solution against water using SnakeSkin™ Dialysis Tubing (MWCO 7 kD) for ~18 hours. The proteins were then lyophilized and stored at -20 °C.

4. Supplementary Tables

Table S1. Plasmids used to express different constructs.

Construct	Expressed from the vector[a]	Essential features of the vector
V_{40}-Tag	pET24a(+)_VT	Kan^r, pBR322 Ori, bicistronic T7 promotors
V_{40}-Tag-S_{60}	pET24a(+)_VTS	
V_{40}-Catcher	pET24a(+)_VC	Kan^r, pBR322 Ori, bicistronic T7 promotors
	pACYCDuet-1_VC[b]	Cm^r, p15A Ori, bicistronic T7 promotors
M–V_{40}-Tag	pETDuet-1_NMT_rs-VT	Amp^r, pBR322 Ori, bicistronic T7 promotors
M–V_{40}-Tag-S_{60}	pETDuet-1_NMT_rs-VTS	
M–V_{40}-Catcher	pETDuet-1_NMT_rs-VC	

[a] rs: The peptide substrate for the NMT enzyme, which is the site of lipidation. [b] This plasmid is used for recombinant expression of MMC using a one-pot approach (Figure S4).

Table S2. DLS results derived from the analysis of autocorrelation functions using cumulants methods.

Temp. (°C)	V_{40}-Tag		M–V_{40}-Tag		V_{40}-Catcher		M–V_{40}-Catcher		V_{40}-Tag-S_{60}		M–V_{40}-Tag-S_{60}		NNC		MNC		NMC		MMC	
	Z_{avg} (SD)[a]	PDI (SD)[b]	Z_{avg}	PDI	Z_{avg}	PDI	Z_{avg}	PDI	Z_{avg}	PDI	Z_{avg}	PDI	Z_{avg}	PDI	Z_{avg}	PDI	Z_{avg}	PDI	Z_{avg}	PDI
**15**	54 (2)	0.6 (.0)	48 (1)	0.6 (.0)	39 (1)	0.4 (.0)	114 (1)	0.3 (.0)	18 (0)	0.5 (.0)	109 (1)	0.4 (.0)	15 (0)	0.3 (.0)	49 (0)	0.2 (.0)	52 (1)	0.2 (.0)	39 (0)	0.2 (.0)
**20**	53 (1)	0.6 (.0)	43 (1)	0.6 (.0)	39 (1)	0.4 (.0)	112 (0)	0.3 (.0)	16 (1)	0.5 (.0)	104 (2)	0.4 (.0)	15 (0)	0.3 (.0)	48 (0)	0.2 (.0)	50 (0)	0.2 (.0)	40 (1)	0.2 (.0)
**25**	177 (26)	0.2 (.0)	51 (8)	0.6 (0.1)	40 (1)	0.4 (.0)	113 (0)	0.3 (.0)	n.d.	n.d.	97 (1)	0.4 (.0)	16 (1)	0.3 (.0)	47 (1)	0.2 (.1)	50 (1)	0.3 (.1)	47 (3)	0.2 (.0)
**30**	222 (4)	0.2 (.0)	2442 (249)	1 (.0)	40 (0)	0.4 (.0)	116 (0)	0.3 (.0)	19 (0)	0.5 (.0)	95 (1)	0.3 (.0)	16 (0)	0.3 (.0)	47 (1)	0.2 (.0)	49 (1)	0.2 (.0)	68 (8)	0.1 (.0)
**35**	233 (2)	0.2 (.0)	3580 (840)	1 (.1)	40 (1)	0.4 (.1)	113 (1)	0.3 (.0)	n.d.	n.d.	93 (3)	0.3 (.0)	15 (1)	0.3 (.0)	48 (1)	0.2 (.0)	49 (1)	0.2 (.0)	77 (1)	0.1 (.0)
**40**	242 (4)	0.1 (.0)	6030 (290)	0.9 (.0)	47 (3)	0.3 (.0)	124 (3)	0.3 (.0)	19 (1)	0.5 (.0)	106 (2)	0.2 (.0)	15 (0)	0.3 (.0)	53 (1)	0.1 (.0)	60 (7)	0.2 (.1)	79 (1)	0.1 (.0)
**45**	1000 (114)	0.1 (.1)	8762 (1238)	1 (.0)	135 (5)	0.3 (.0)	146 (0)	0.3 (.0)	25 (1)	0.7 (.0)	109 (1)	0.2 (.0)	15 (0)	0.2 (.0)	78 (11)	0.3 (.1)	194 (60)	0.5 (.0)	81 (0)	0.1 (.0)
**50**	1990 (126)	0.3 (.1)	12500 (1710)	1 (.1)	184 (1)	0.3 (.0)	184 (35)	0.5 (.0)	42 (1)	0.2 (.0)	114 (2)	0.2 (.0)	35 (0)	0.0 (.0)	109 (10)	0.7 (.0)	335 (89)	0.6 (.1)	81 (0)	0.1 (.0)
**55**	2650 (126)	0.4 (.1)	12900 (941)	1 (.0)	85 (9)	0.3 (.1)	378 (33)	0.7 (.1)	45 (0)	0.0 (.0)	124 (2)	0.3 (.0)	43 (0)	0.0 (.0)	110 (6)	0.7 (.1)	929 (154)	0.8 (.2)	81 (0)	0.1 (.0)
**60**	3150 (198)	0.6 (.1)	13100 (1930)	1 (.0)	92 (3)	0.5 (.0)	475 (31)	0.7 (.0)	47 (0)	0.0 (.0)	347 (69)	0.4 (.0)	45 (0)	0.0 (.0)	100 (8)	0.7 (.1)	1020 (324)	1 (.3)	81 (1)	0.1 (.0)
**65**	3790 (371)	0.7 (.1)	21300 (5290)	0.9 (.2)	89 (5)	0.5 (.0)	400 (7)	1 (.0)	48 (0)	0.0 (.0)	1360 (167)	0.7 (.0)	45 (0)	0.0 (.0)	95 (2)	0.7 (.0)	973 (127)	0.8 (.1)	81 (0)	0.1 (.0)

a – Average hydrodynamic diameter (nm) derived from the cumulants method (n = 3); b – Polydispersity index derived from the cumulants method, (n = 3); n.d. – not determined

Table S3. Size distributions for constructs forming worm-like micelles derived from the analysis of TEM images.

Constructs	T,°C	Length, nm mean ± SD (*n*)	Core, nm mean ± SD (*n*)	Width, nm mean ± SD (*n*)
**M-V_{40}-Tag**	40	3051 ± 1140[a] (63)	n.d.	38 ± 10 (61)
**M-V_{40}-Tag-S_{60}**	40	495 ± 326 (359)	9 ± 2 (89)	55 ± 11 (87)
**M-V_{40}-Catcher**	20	123 ± 85 (216)	7 ± 3 (53)	49 ± 10 (60)
	40	129 ± 89 (384)	8 ± 3 (65)	44 ± 9 (84)
**MNC**	40	261 ± 172 (57)	12 ± 3 (54)	77 ± 20 (54)
**NMC**	40	81 ± 28 (77)	22 ± 6 (51)	73 ± 19 (51)
**MMC**	20	125 ± 49 (290)	7 ± 3 (237)	39 ± 11 (148)
	40	169 ± 61 (261)	10 ± 3 (224)	33 ± 8 (241)

[a] – Most fibers extended beyond the imaging window. Maximum observable length measured is reported. n.d. – not determined due to the lack of contrast between the core and corona of these structures.

Table S4. Definitions and categorization of molecular features extracted from MD simulations as PCA input.

	Parameter[a]	Unit	Definition	Category
1	<VC-VT>	Å	Root-mean-square end-to-end distance between VC and VT blocks	Form
2	<VC-S>	Å	Root-mean-square end-to-end distance between VC and S blocks	Form
3	<VT-S>	Å	Root-mean-square end-to-end distance between VT and S blocks	Form
4	R_g (VC)	nm	Radius of gyration of VC block	Size
5	R_g (C)	nm	Radius of gyration of branching point (SpyCatcher-Tag complex)	Size
6	R_g (VT)	nm	Radius of gyration of VT block	Size
7	R_g (S)	nm	Radius of gyration of S block	Size
8	V_C <W>	N/A	average number of water molecules in the first hydration layer of V_C	hydration
9	V_C <HB>	N/A	average number of hydrogen bonds (HB) between the V_C and water	hydration
10	C <W>	N/A	average number of water molecules in the first hydration layer of branching point	hydration
11	C <HB>	N/A	average number of HB between the branching point and water	hydration
12	VT <W>	N/A	average number of water molecules in the first hydration layer of V_T	hydration
13	VT <HB>	N/A	average number of HB between the VT and water	hydration
14	S <W>	N/A	average number of water molecules in the first hydration layer of S	hydration
15	S <HB>	N/A	average number of HB between S and water	hydration

[a] – VC: The V_{40} linearly fused to the Catcher domain; VT: V40 linearly fused to the Tag domain; S: S_{60}; C: refers to the branching point formed after the reaction between SpyCatcher and SpyTag.

5. Supplementary Figures

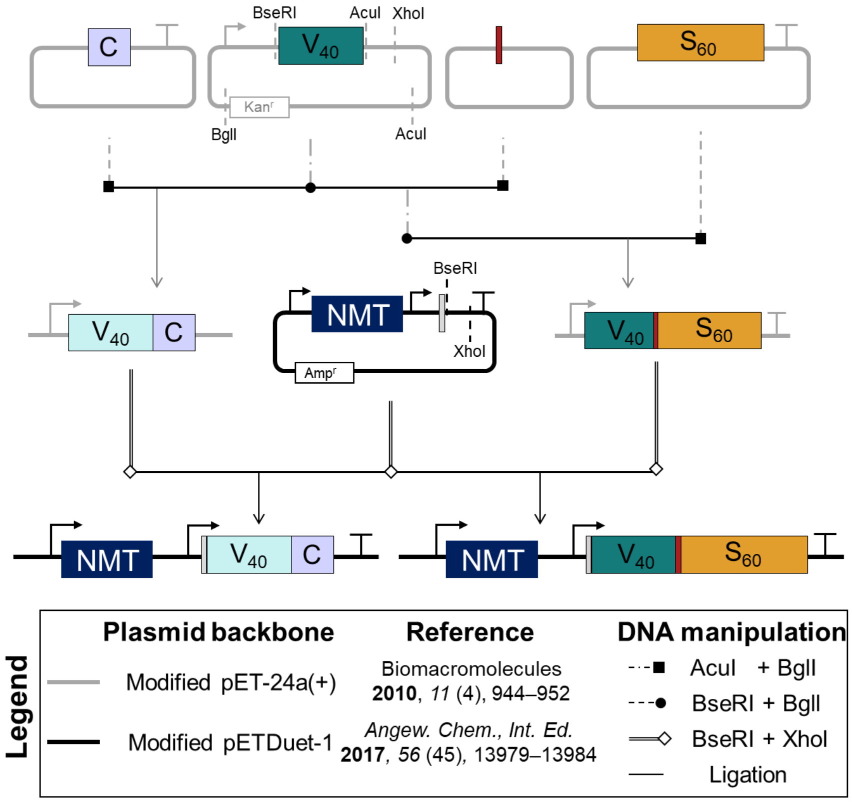

Figure S1. A schematic of cloning steps used to construct plasmids to produce SAFE amphiphiles and the linear controls used. The genes encoding the main building blocks (V_{40}, S_{60}, SpyCatcher, and SpyTag) were cloned into pET-24a(+) plasmids. Recursive directional ligation was used to assemble the genes for fusion proteins in the desired order. The assembled gene was then subcloned into pETDuet-1 vectors. These bicistronic vectors were used to co-express the NMT enzyme and the desired protein fused to NMT peptide substrate to produce myristoylated proteins in *E.coli*.

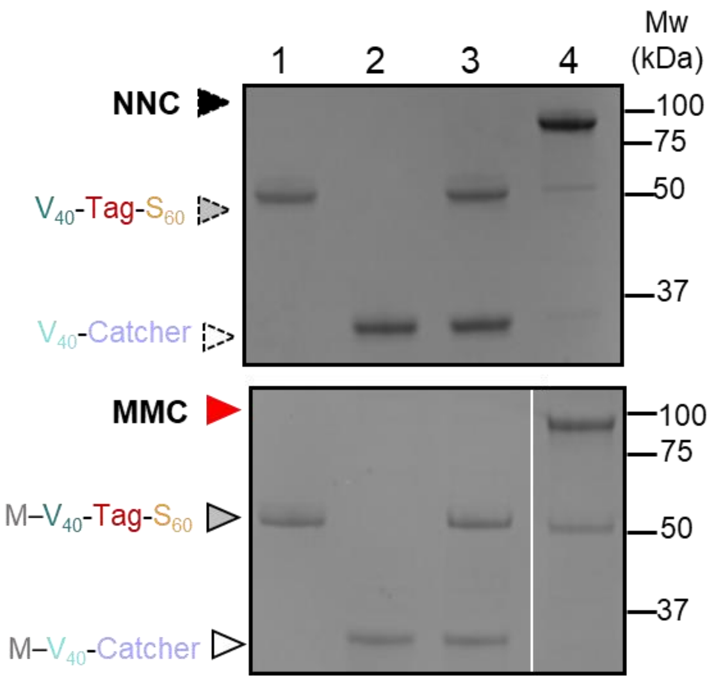

Figure S2. Monitoring isopeptide formation reactions of unmodified and myristoylated linear building blocks using SDS-PAGE. In each panel, lanes 1 and 2 are starting materials—the ELP diblock copolymer, (M)-$(GVGVP)_{40}$-Tag-$(GSGVP)_{60}$, and ELP fused to Catcher, (M)-$(GVGVP)_{40}$-Catcher. Lanes 3 and 4 are reaction mixtures at time 0 and 2 h. Myristoylation did not alter the reactivity of Spy pairs under these conditions.

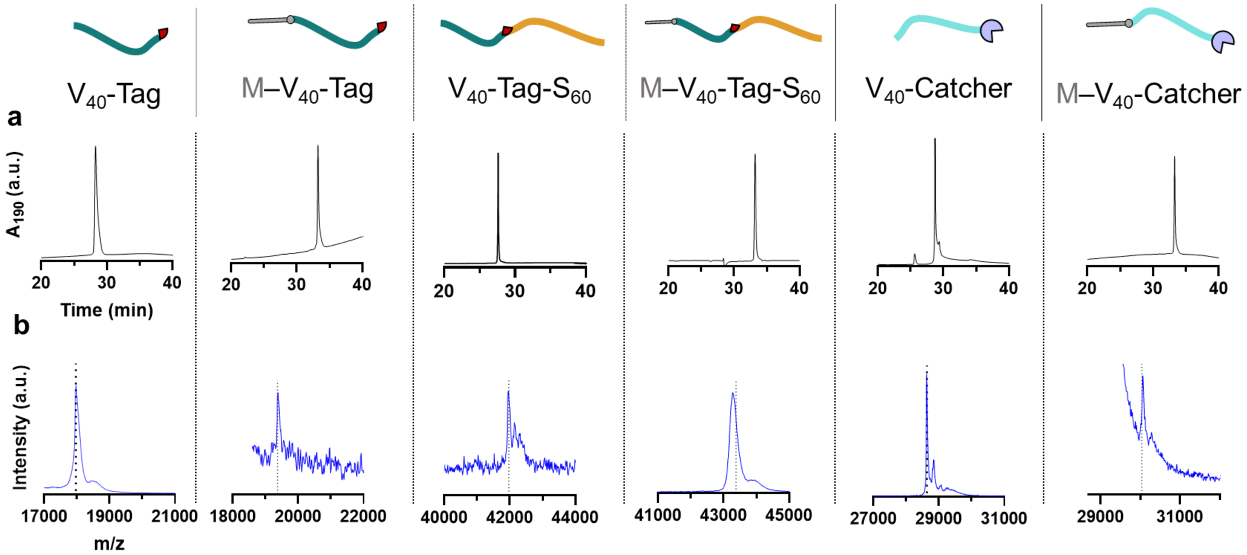

Figure S3. Molecular characterization of purified linear building blocks (and controls). **(a)** Analytical RP-HPLC of each component. Modification with the hydrophobic myristoyl group increased the retention time of the protein. V_{40}-Tag and M–V_{40}-Tag were analyzed on C4 columns because of their high hydrophobicity. All other constructs were analyzed using a C18 column. **(b)** MALDI-TOF-MS analysis of proteins. Modification with myristic acid (and removal of water) increased the m/z ratio by 210. The vertical dotted line in b denotes the theoretical (calculated) molecular weight of each construct.

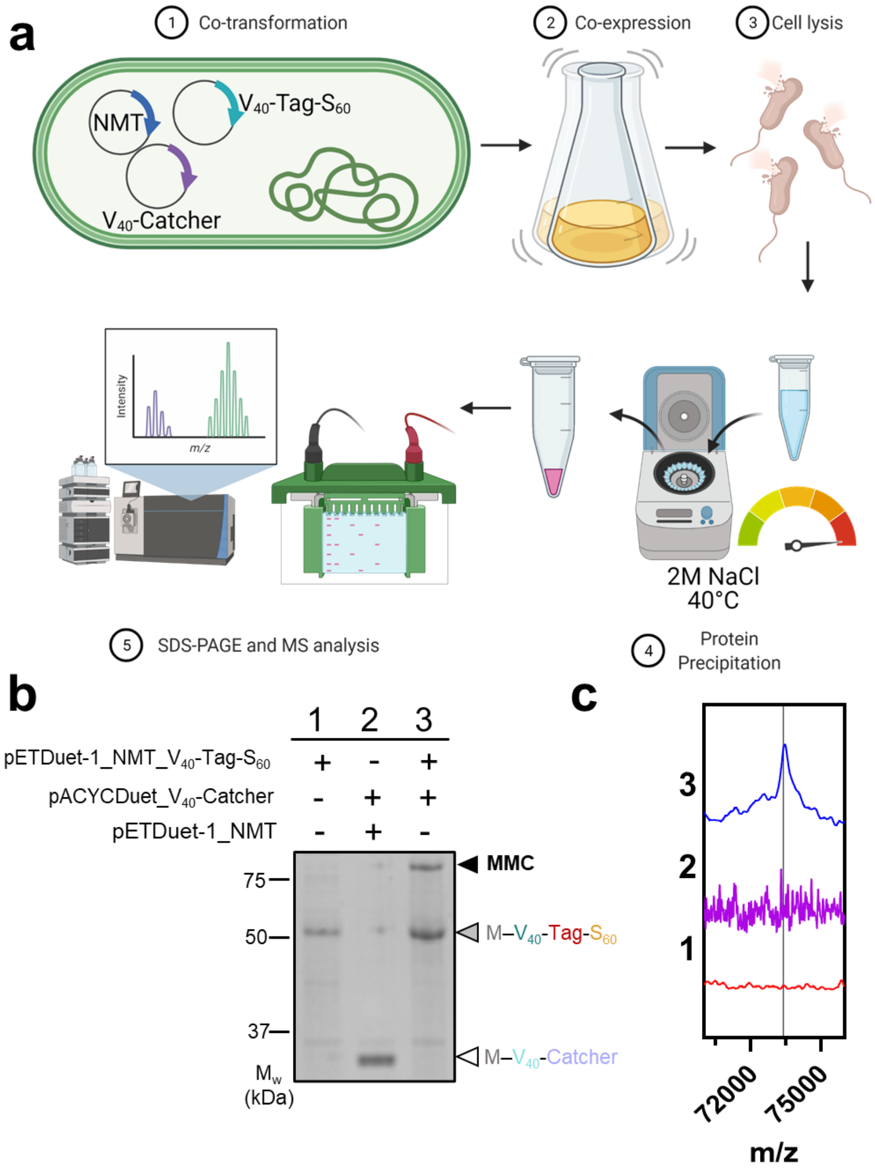

Figure S4. SAFE amphiphiles produced using a one-pot recombinant expression, tandem PTM process. **(a)** A schematic of the proof-of-concept experiment using orthogonal plasmids (with compatible origins of replication and antibiotic selection markers) for co-expression of NMT, V40-Tag-S60, and V40-Catcher proteins in one cell. After cell lysis, phase separation of all proteins fused to ELP domains was triggered by the addition of kosmotropic salts at 40 °C. The protein pellet was separated from the supernatant and redissolved in water:ethanol mixture (1:1 v/v) for analysis using SDS-PAGE and MALDI. **(b)** The presence of both SpyCatcher and SpyTag are required for branching PTM (cf. lane 3 with lanes 1 and 2). **(c)** MALDI-TOF-MS was used to confirm the molecular weight of MMC produced in the one-pot reaction. Figure S4a is created with BioRender.com.

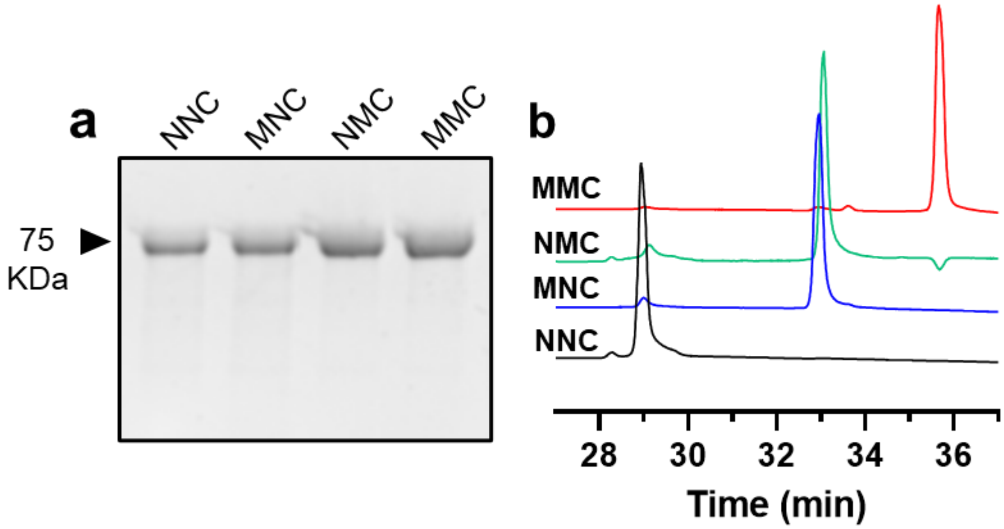

Figure S5. The purity of SAFE amphiphiles was confirmed by SDS-PAGE **(a)** and analytical reverse-phase HPLC **(b)**.

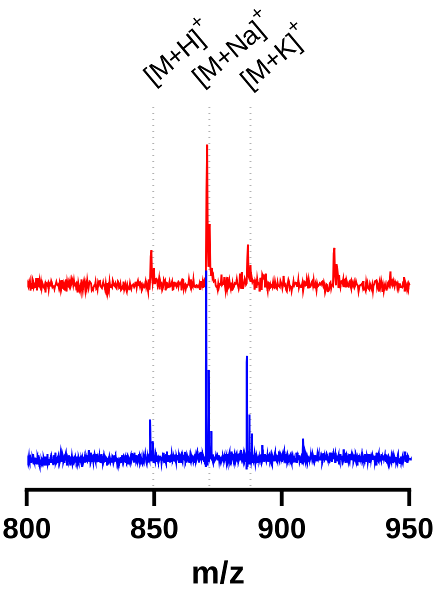

Theoretical Mw = 848.35 Da
Observed m/z
$[M+H]^+$ = 849.33 Da
$[M+Na]^+$ = 870.83 Da
$[M+K]^+$ = 887.50 Da

Figure S6. The N-terminal myristoylation was confirmed by digestion of the proteins with trypsin and the analysis of peptide fragments using MALDI-TOF-MS. The N-terminal glycines of V_{40}-Tag-S_{60} (red) or V_{40}-Catcher expressed in the presence of NMT were myristoylated.

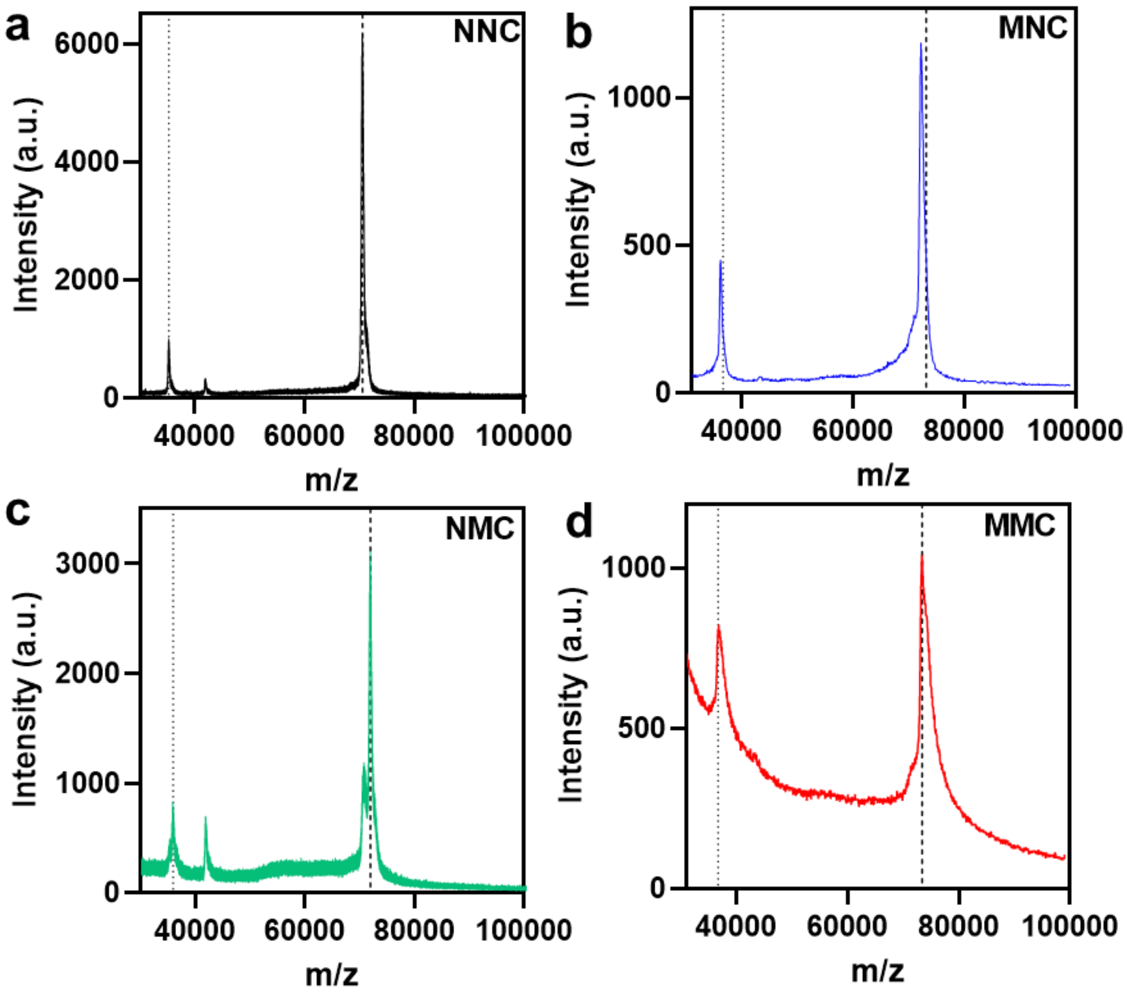

Figure S7. MALDI-TOF-MS analysis of SAFE amphiphiles: (a) NNC, (b) MNC, (c) NMC, and (d) MMC. The vertical dashed and dotted lines are drawn to denote the theoretical (calculated) $[M+H]^+$ and $[M+2H]^{2+}$, respectively.

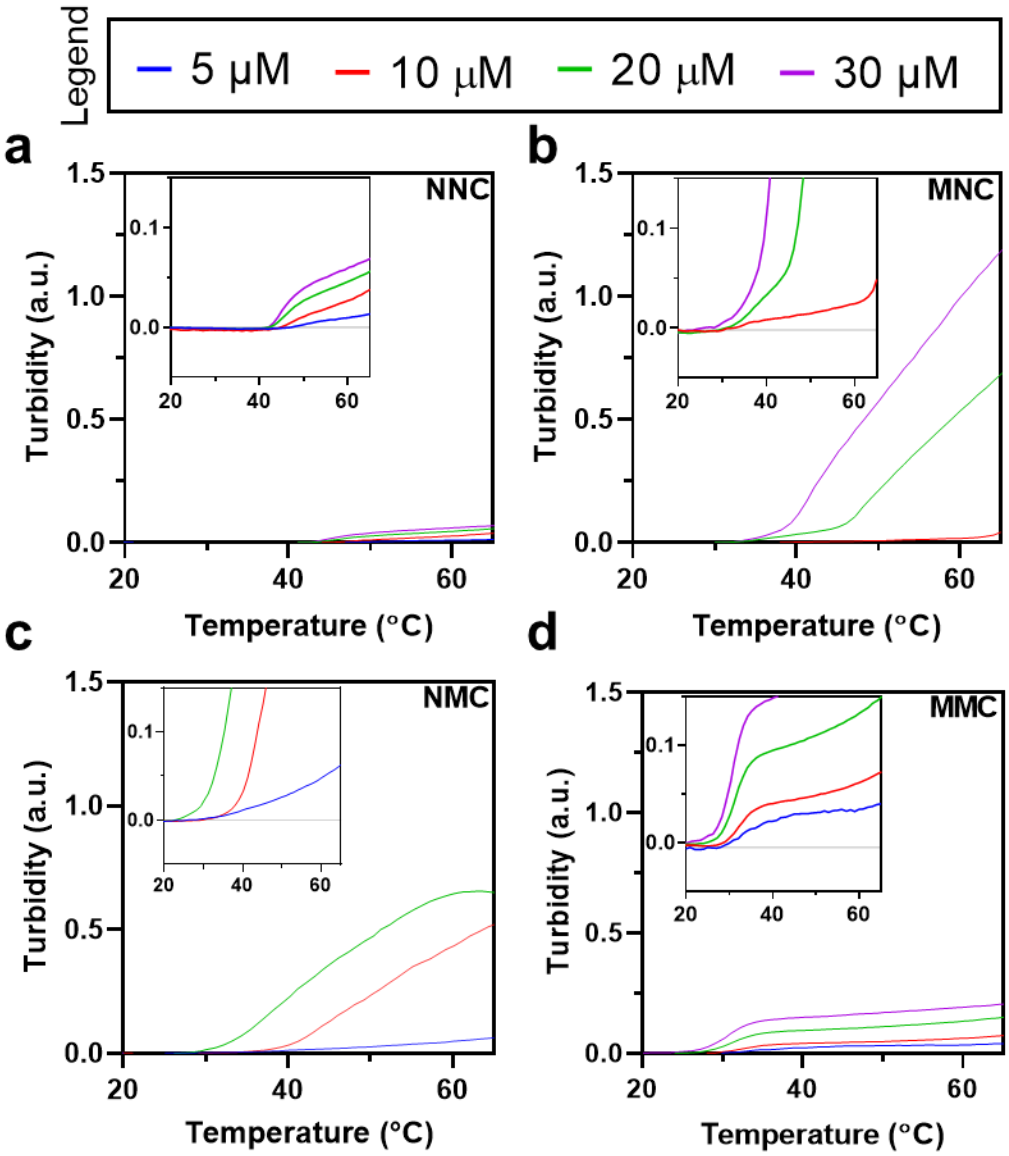

Figure S8. Turbidity profiles of star amphiphiles at different concentrations (5–30 µM in PBS). The inset depicts the evolution of the turbidity profile close to the observed transition temperatures. The turbidity of both NNC and MMC solutions only increased modestly with temperature (a, d), while the turbidity of single-lipidated MNC and NMC solutions (b and c), increased significantly above transition temperature. The phase-behavior of single-lipidated constructs varied significantly with their concentration.

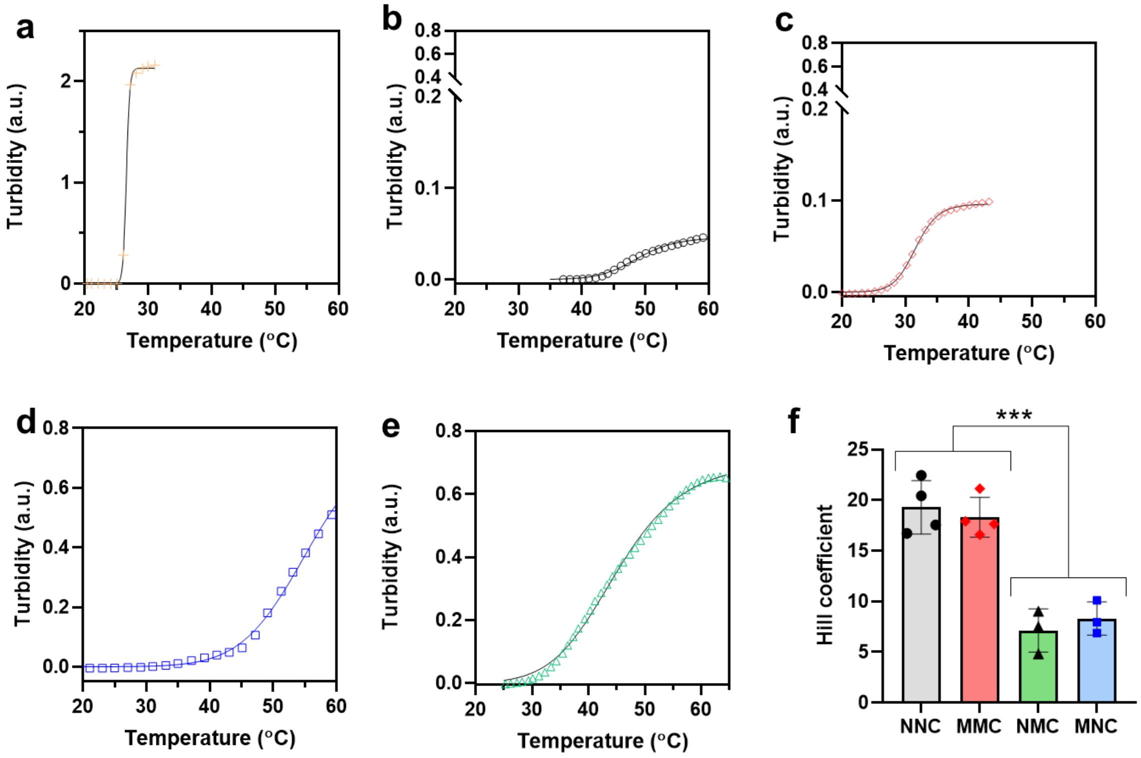

Figure S9. The analysis of the turbidity profiles using Hill saturation function. (a) M-V_{40}-Tag; **(b)** NNC; **(c)** MMC; **(d)** MNC; **(e)** NMC. **(f)** Bar plot summarizing the Hill coefficients derived from the turbidity plots of various constructs. Hill coefficients (slope) are derived by fitting the turbidimetry plots to the following equation, Abs = Abs(Max) × $T^h/(T_{1/2}^h + T^h)$, where h is the Hill slope, $T_{1/2}$ is the temperature corresponding to the midpoint (which is close to T_t). A larger number signifies higher cooperativity in the phase behavior of the systems. Star amphiphiles exhibited lower Hill coefficients compared to lipidated linear control. Single-lipidated constructs have lower Hill coefficients compared to non-lipidated or double lipidated constructs. One-way ANOVA was used with Turkey HSD post-hoc test for comparisons between different groups. **: p <0.001. The fitted data for [protein] = 20 μM in PBS is provided as representative examples in a–e. Please note that the Y-axes are plotted using different scales because of observed differences in the AU_{max} for each construct.

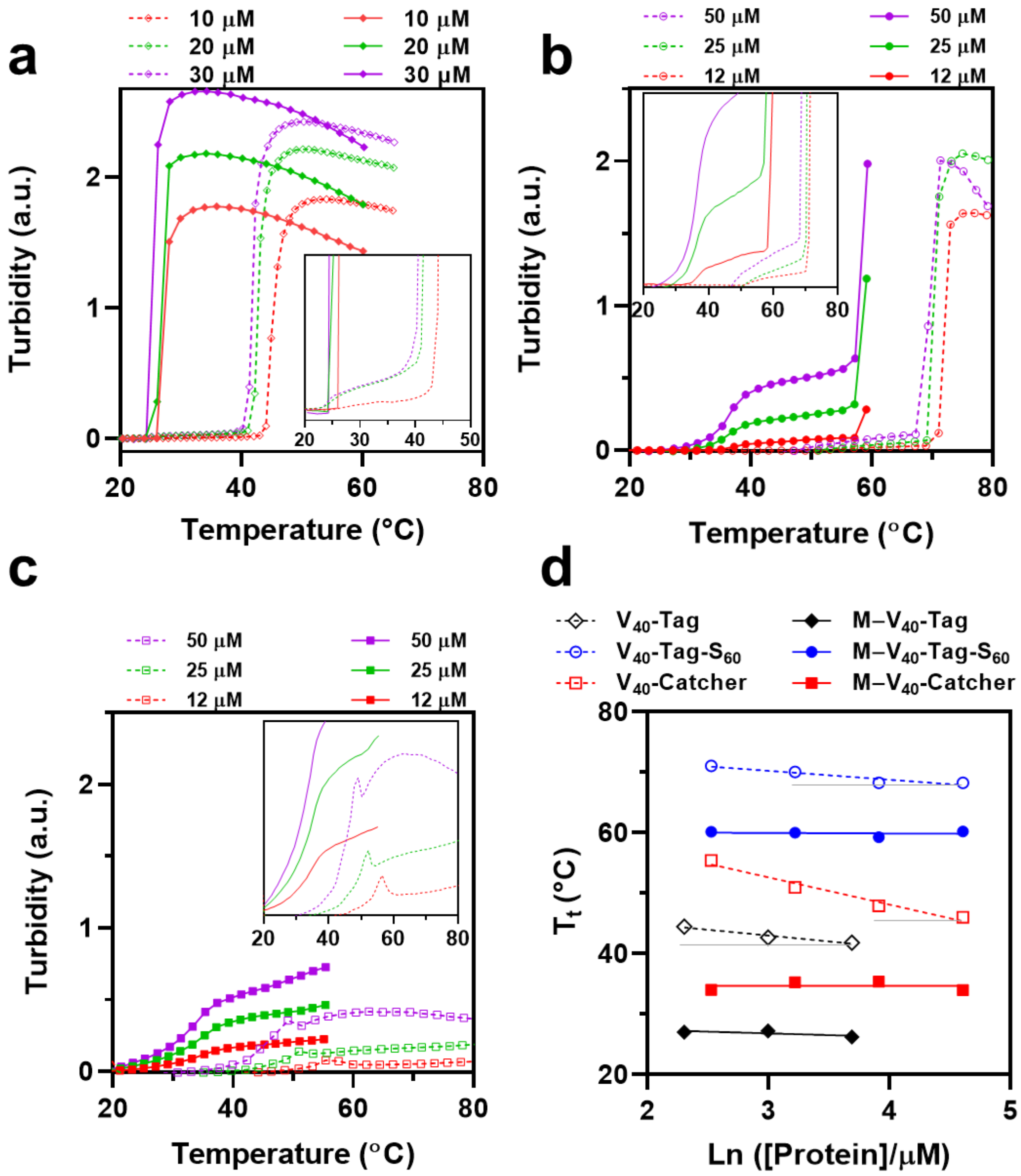

Figure S10. Characterization of thermoresponse (and its concentration dependence) for linear controls in PBS using turbidimetry. **(a)** V_{40}-Tag and M–V_{40}-Tag; **(b)** V_{40}-Tag-S_{60} and M–V_{40}-Tag-S_{60}; **(c)** V_{40}-Catcher and M–V_{40}-catcher; **(d)** The concentration dependence of linear constructs transition temperatures. In all panels, nonmyristoylated samples are represented with open symbols (and dashed lines), while lipidated controls are shown using filled symbols (and solid lines).

The T_t of nonmyristoylated constructs (V_{40}-Tag, V_{40}-Catcher, and V_{40}-Tag-S_{60}) exhibited stronger concentration dependence. The horizontal grey line is added to schematically convey the magnitude of change as a function of temperature. The phase-behavior of myristoylated constructs was less sensitive to their solution concentration, while the order of transition temperatures was consistent with the overall hydrophilicity of constructs. Two deviations from these general trends are noted: 1) Similar to MNC, V_{40}-Tag and M–V_{40}-Tag-S_{60} exhibited two distinct transition temperatures. We attribute the lower transition temperature to the formation of nano-assemblies from unimers/oligomers, and the higher transition temperature to bulk phase separation. The second T_t is plotted in d. 2) The turbidity profile of V_{40}-Catcher exhibited a noticeable peak as a function of temperature, i.e., turbidity initially increased followed by a noticeable decrease as the temperature was raised above T_t (see inset of c). This was likely due to changes in hydration of oligomers as temperature was increased (see Figure S14c for DLS).

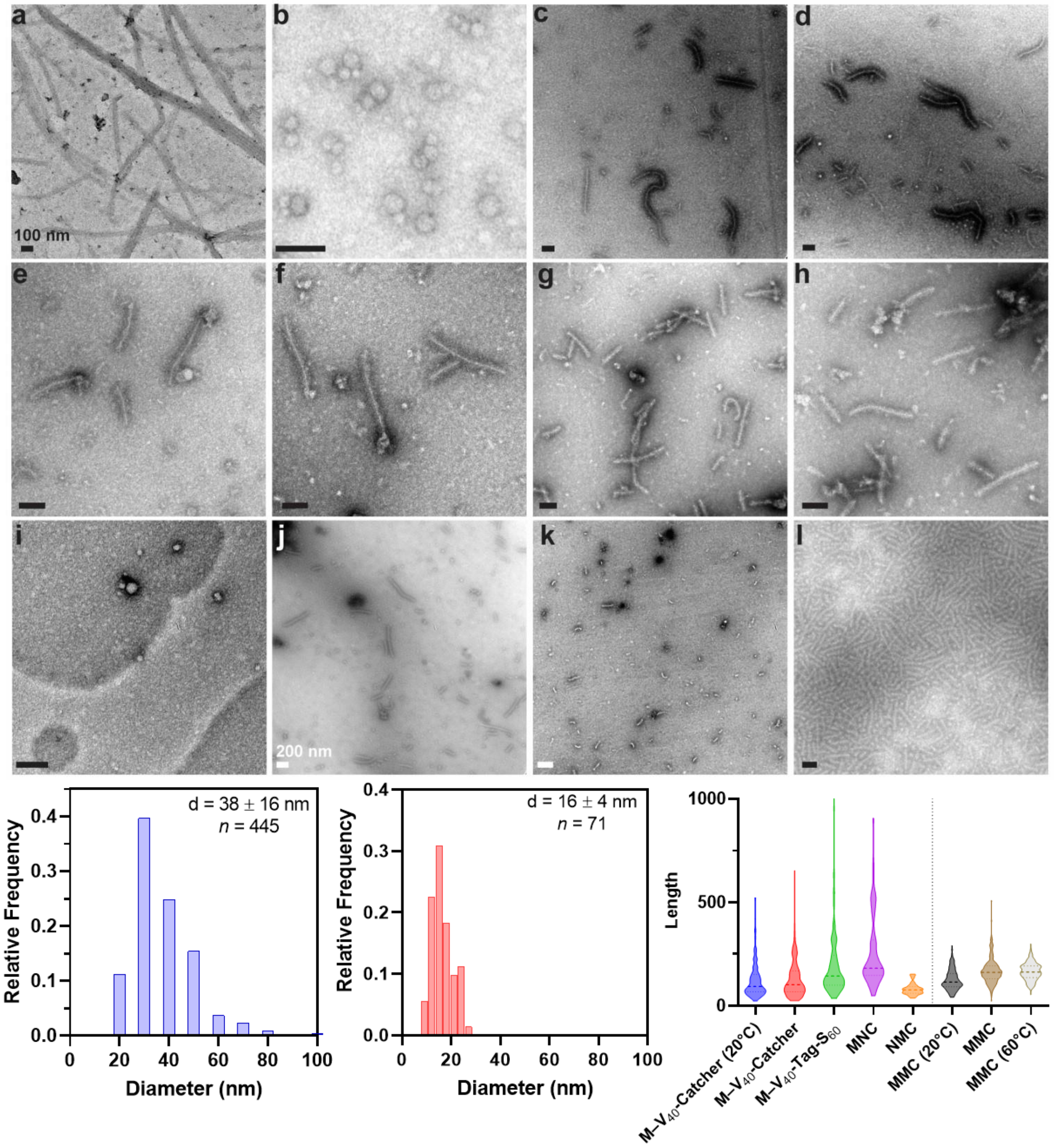

Figure S11. Representative TEM images for linear (a–h) and star amphiphiles (i–l), and the select statistical size distributions derived from image analysis (m-o). **(a)** M-V_{40}-Tag formed long fibers at 40 °C. M-V_{40}-Tag-S_{60} formed spherical micelles at 20 °C **(b)**, and a polydisperse mixture of worm-like micelles at 40 °C **(c, d)**. M-V_{40}-Catcher formed a polydisperse mixture of worm-like micelles at 20 °C **(e, f)** and 40 °C **(g, h)**. NNC formed spherical micelles at 60 °C (**i**). Both MNC and NMC formed worm-like micelles at 40 °C (**j, k**), but the morphology of these micelles differs based on the location of the attached lipid. MNC forms polydisperse worm-like materials (WLMs) with canonical cylindrical morphology. However, NMC forms shorter micelles with noticeably larger cores (visualized as the white area in the stained images). Both constructs formed coacervates at elevated temperatures. **(l)** MMC formed stable nanoworms with narrow polydispersity at 40 °C. (**m, n**) The size-distribution histograms for the diameter of spherical particles formed by M–V_{40}-Tag-S_{60} (at 20 °C) and NNC (at 60 °C). Measurement results are reported as mean ± SD. **(o)** A violin plot showing the length-distribution of constructs that formed anisotropic worm-like micelles. Unless specified, the sample temperature is 40 °C. The horizontal dashed line denotes the median value.

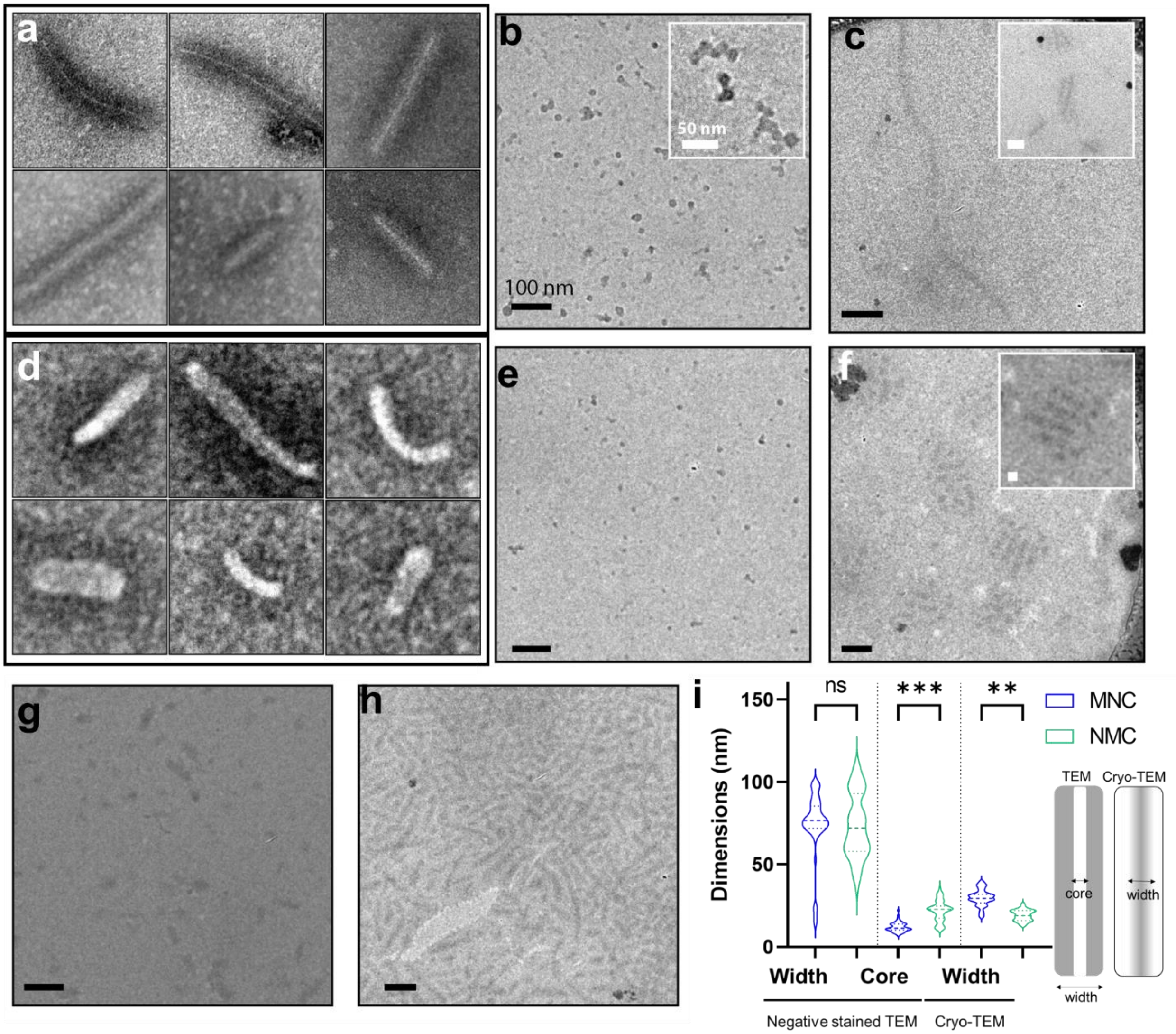

Figure S12. The Visualization of single-lipidated constructs using negatively stained and cryo-TEM. (a) Magnified TEM images of MNC at 40 °C. Cryo-TEM images of MNC **(b,c)**, NMC **(e,f)**, and MMC **(g,h)** below and above LCST. **(i)** Violin plots summarizing the size-distributions of single-lipid nano-assemblies obtained from the analysis of electron microscopy images. The graphic is a schematic of the contrast between different segments of the assemblies in stained and cryo-TEM. One-way ANOVA was used with Sidak post hoc tests for comparisons between different groups. *p*-value summary ns: >0.05, ***: <0.001, ** <0.01.

We note that cryo-TEM and stained TEM visualize different segments of SAFE assemblies. The contrast in negatively stained TEM is due to the differential binding or partitioning of staining agent (uranium acetate) while the changes in the hydration level are the source of the contrast in cryo-TEM images (Figure S12i). Therefore, the width of the assemblies in cryo-TEM likely represents the hydrophobic core of these assemblies as the ELP chains often remain hydrated and are not visualized. These two complementary imaging techniques provide additional insights into the structural hierarchy of SAFE assemblies.

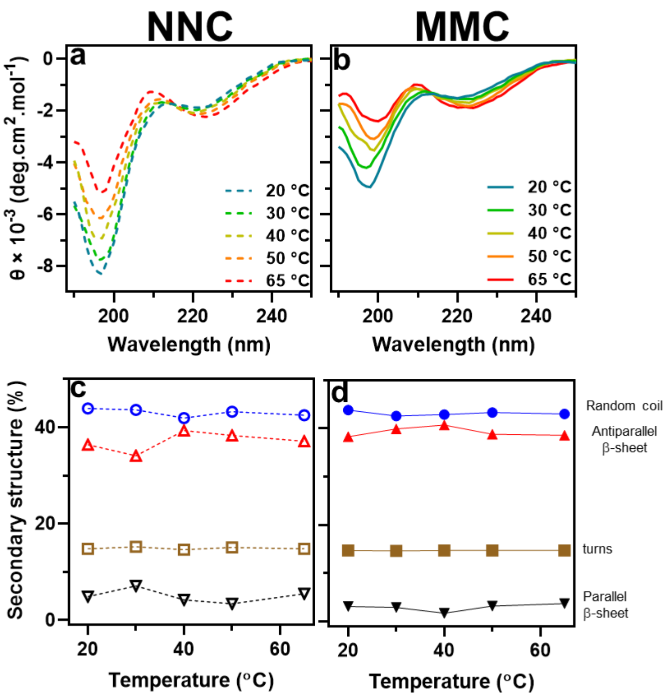

Figure S13. Variable-temperature circular dichroism signal of NNC **(a)** and MMC **(b)**, and the secondary structure elements content derived from the deconvolution of CD signals for each construct **(c,d)**. Both NNC and MMC exhibit the characteristic negative CD signal at ~200 nm and ~220 nm, corresponding to disordered domains of ELP and β-sheets within the CnaB2 domain of Catch-Tag complex (branching point). The CD signal did not change significantly as the temperature was increased to 65 °C, as only a modest decrease in the disordered signal (200 nm) was observed. This is consistent with the reported thermal stability of CnaB2 domain[5] as well as the stability of SAFE's nanostructures reported in our work.

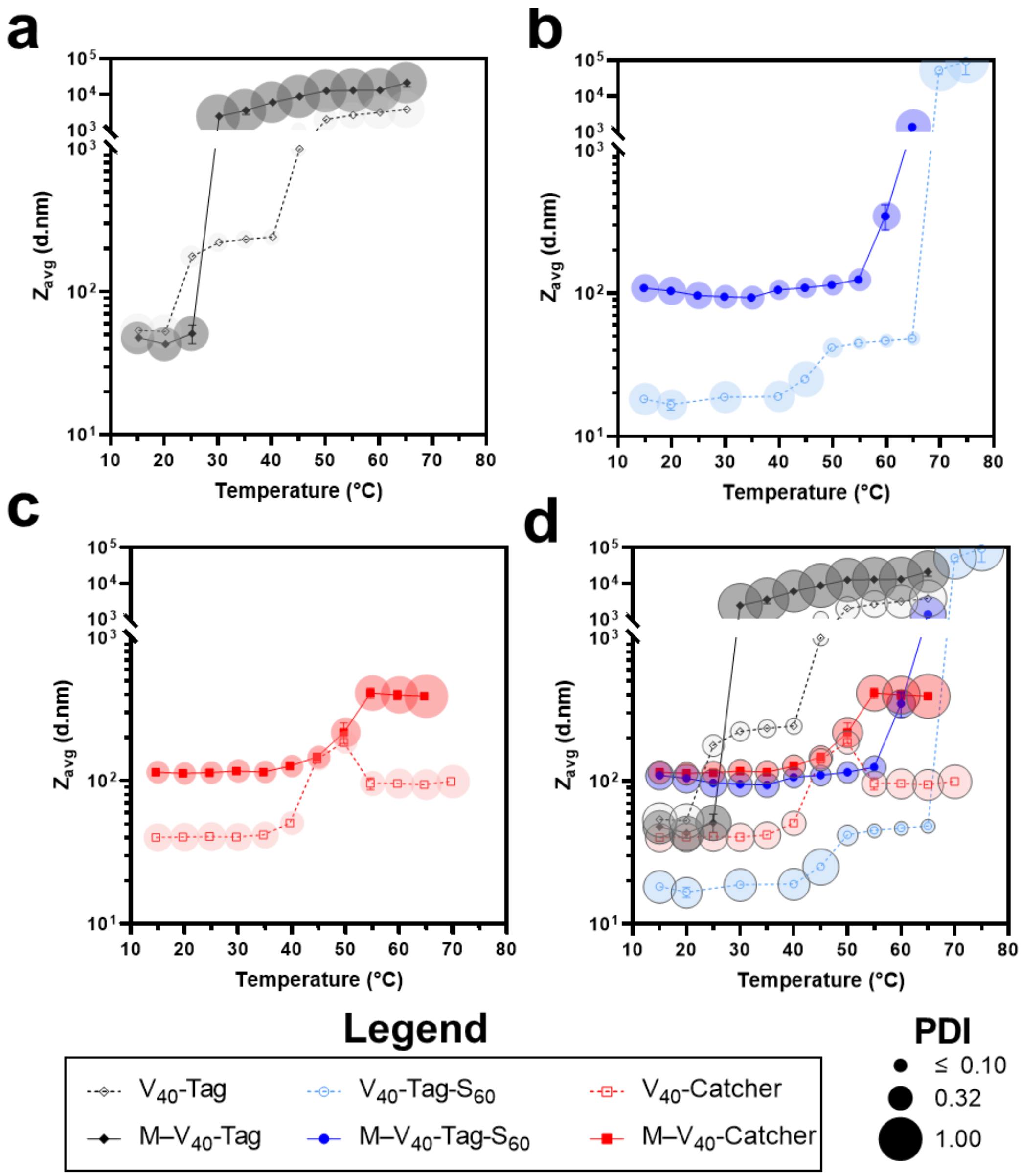

Figure S14. Dynamic light scattering analysis of the temperature-dependent assembly of linear controls. A bubble plot summarizing the size of aggregates derived from the cumulant method, **(a)** V_{40}-Tag and M–V_{40}-Tag; **(b)** V_{40}-Tag-S_{60} and M–V_{40}-Tag-S_{60}; **(c)** V_{40}-Catcher and M–V_{40}-Catcher; and **(d)** superimposition of data presented in a–c. The symbol in the center of each bubble represents the average hydrodynamic radius (Z_{avg}), while the area represents the polydispersity index (PDI). In all panels, nonmyristoylated samples are represented with open symbols (and dashed lines), while lipidated controls are shown using filled symbols (and solid lines). Lines are added as a visual reference. All proteins were analyzed at 20 µM in PBS, except M-V_{40}-Tag (30 µM in PBS). Error bars are standard deviations of three measurements.

Myristoylation generally increased the size of assemblies at T < T_t and reduced T_t. Above T_t, most constructs formed large micron size coacervates, except for proteins fused to Catcher domain (cf. panels a and b with c). The fusion of Catcher protein stopped the formation of micron size aggregates even at elevated temperatures. Consistent with the turbidimetry, DLS of V_{40}-Catcher also showed a noticeable peak ~ 50 °C. All samples exhibited broad polydispersity except for V_{40}-Tag-S_{60} between 50–70 °C, an observation that is consistent with the formation of spherical micelles once the temperature is increased above the transition temperature of hydrophobic block.

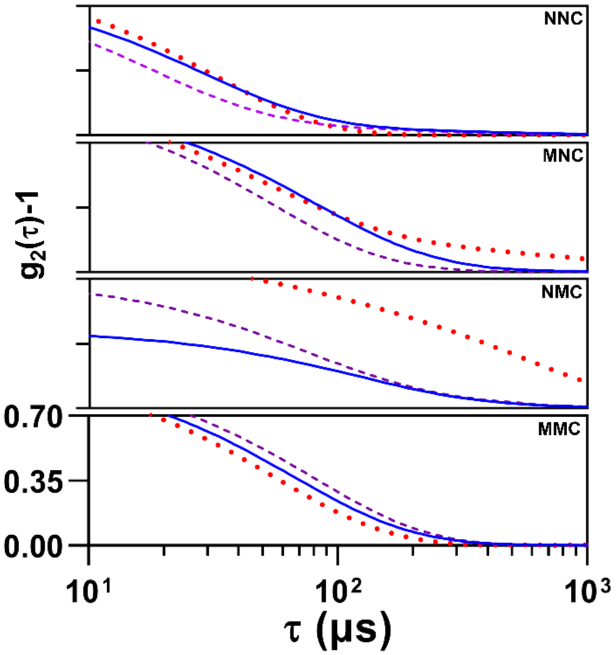

Figure S15. Dynamic light scattering confirms that lipidation pattern modulates the temperature-dependent assembly of star amphiphiles. Autocorrelation functions of each construct dissolved in PBS (20 μM) at 20 °C (blue solid line), 40 °C (purple dashed line), and 60 °C (red dotted line).

In DLS experiments, the detector quantifies the fluctuations of the scattered light, which are caused by the motion of molecules and particles in solution. The autocorrelation function (ACF) is the mathematical description of the fluctuations of the scattered light, i.e., it shows how rapidly the scattering intensity fluctuates as the function of time. The comparison between the autocorrelation functions provides a way to assess (compare) the assembly of various constructs without resorting to specific models (e.g., cumulants or CONTIN), which have their own underlying assumptions and simplifications. Importantly, the autocorrelation functions include all the information necessary for subsequent analysis by other researchers (or software suites). The comparison of two key features of ACFs, decay time and mode (i.e., the intersection with x-axis and mono-/bi- phasic decay), confirms that lipidation pattern alters the assembly and temperature-responsiveness of SAFE assemblies without making assumptions about the shape of the aggregates.

At 20 °C, the mono-modal decay time of NNC (t ~10^2 μs) was consistent with the presence of unimeric or unassembled species, while all lipidated constructs formed nanoscale assemblies (10^2 μs < t < 10^3 μs). Consistent with turbidimetry results, the ACFs for NNC and MMC shifted only slightly at higher temperatures (40 and 60 °C), but both constructs remained in the nanoscale range. The behavior of single-lipid constructs was noticeably different, as nanoscale assemblies at 20 °C formed much larger aggregates at 60 °C, t > 10^3 μs consistent with the size regime in the micron range. In addition, we observed subtle differences between the temperature-dependent nano-assembly of NMC and MNC. The NMC assemblies remained in the nanoscale range at 20 and 40 °C and formed larger micron-sized aggregates at 60 °C. In contrast, the ACF of MNC deviated from one-phase exponential decay, which indicates the formation of a mixture of small and large particles.

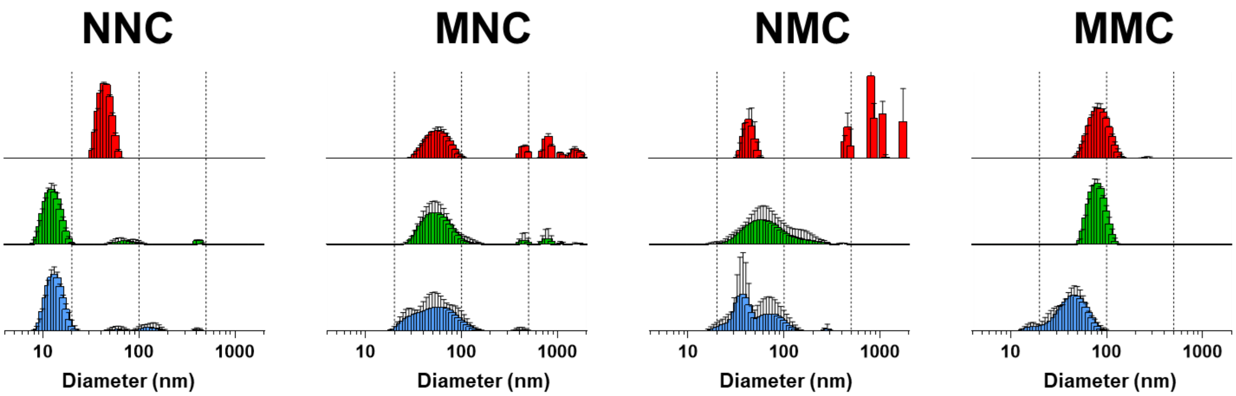

Figure S16. Intensity distributions for star amphiphiles at 20 °C (bottom, blue bars), 40 °C (middle panel, green bars), and 60 °C (top panel, red bars) derived from the analysis of ACFs shown in Figure S15 with the CONTIN algorithm. All proteins were analyzed at 20 μM in PBS. Error bars are standard deviation of three measurements.

NNC mostly remained as unimers at 20 and 40 °C, but formed micellar assemblies with diameter ~40 nm at 60 °C. All myristoylated SAFEs assembled even at 20 °C, forming micelles of ~40–50 nm with relatively broad polydispersity. NMC and MNC formed larger micron size aggregates as the temperature was increased to 60 °C. In contrast, the size of MMC assemblies increased to ~80 nm when the temperature was increased above its T_t, and these assemblies remained stable after heating to 60 °C.

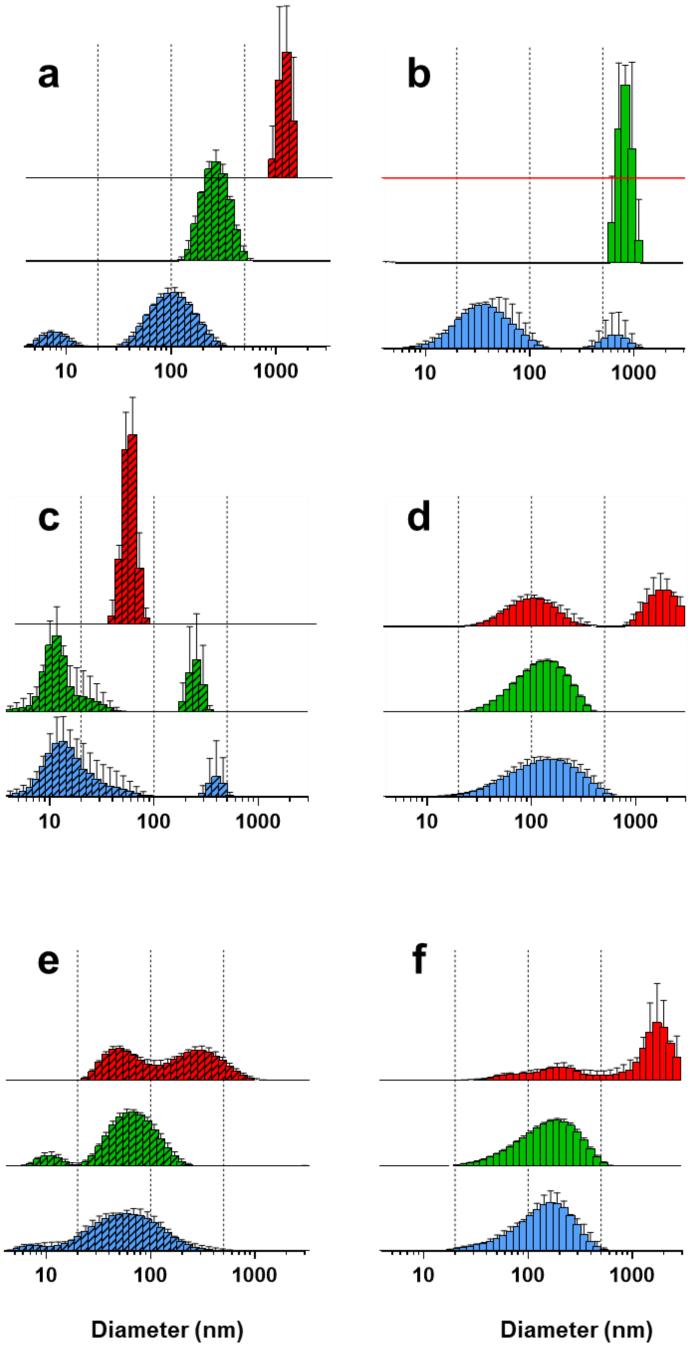

Figure S17. Intensity distributions for linear unmodified (dashed bars) and myristoylated (solid bars) control amphiphiles at 20 °C (bottom, blue bars), 40 °C (middle panel, green bars), and 60 °C (top panel, red bars) derived from the analysis of CRFs with the CONTIN algorithm. **(a,b)** V_{40}-Tag and M–V_{40}-Tag; **(c, d)** V_{40}-Tag-S_{60} and M–V_{40}-Tag-S_{60}; and **(e, f)** V_{40}-Catcher and M–V_{40}-Catcher. The size of M–V_{40}-Tag at 60 °C exceeded the limits of CONTIN algorithm (>10μm). The vertical dashed lines at 20, 100, and 500 nm are added to aid the comparison of plots. All proteins were analyzed at 20 μM in PBS, except M-V_{40}-Tag (30 μM in PBS). Error bars are standard deviation of three measurements.

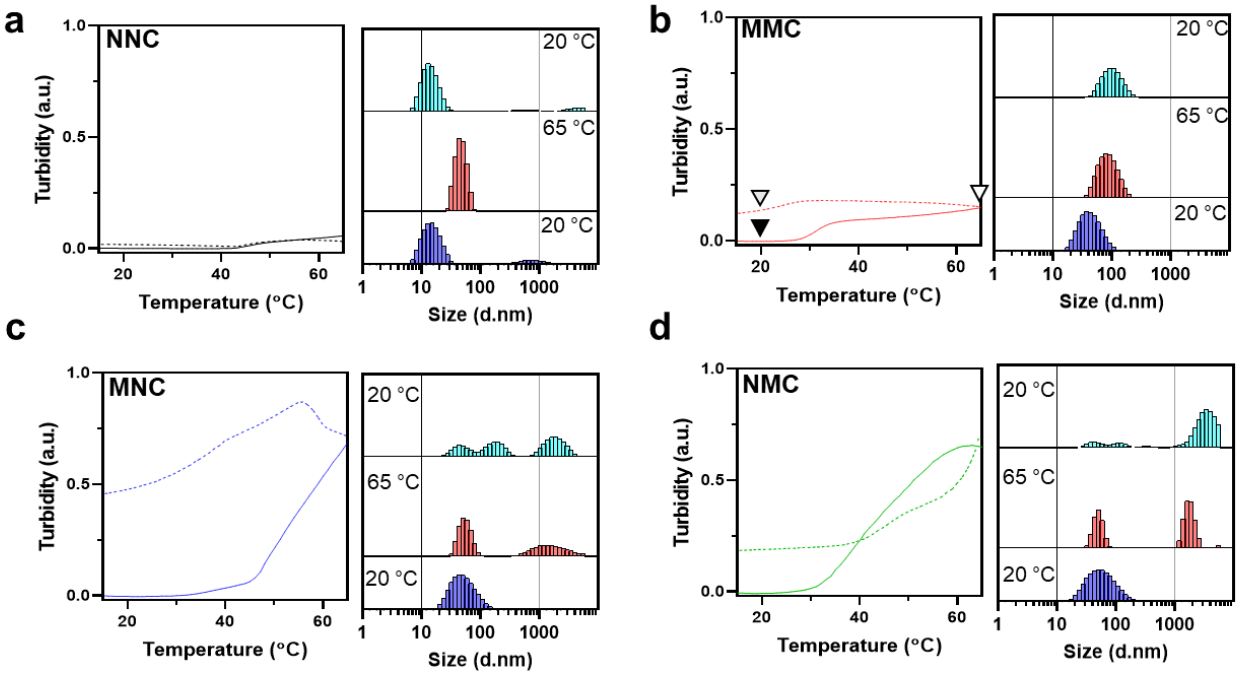

Figure S18. The reversibility of the star amphiphiles' phase behavior and assembly monitored using turbidimetry and DLS. (a) NNC; (b) MMC; (c) MNC; (d) NMC. The non-lipidated NNC exhibit a reversible LCST and self-assembly, while lipidated amphiphiles exhibit different degrees of hysteresis. The coacervates of single-lipidated constructs dissolve when the temperature is reduced below their T_{cp}, but the nano-assembled structure persists in solution. The proteins were heated/cooled between 15-65 °C at a rate of ± 1 °C/min (in turbidimetry) or ± 5 °C increments in DLS. In each panel, the solid and dashed lines correspond to the turbidity of the solution obtained during heating or cooling, respectively. The intensity distributions at 20 °C, 65 °C, and again at 20 °C (blue, red, and cyan bars) are plotted. The arrows in S18b are added to highlight the turbidity of the solution corresponding to these temperatures for MMC. Vertical lines at 10 and 1000 nm are drawn as a visual reference. [protein] = 20 µM in PBS.

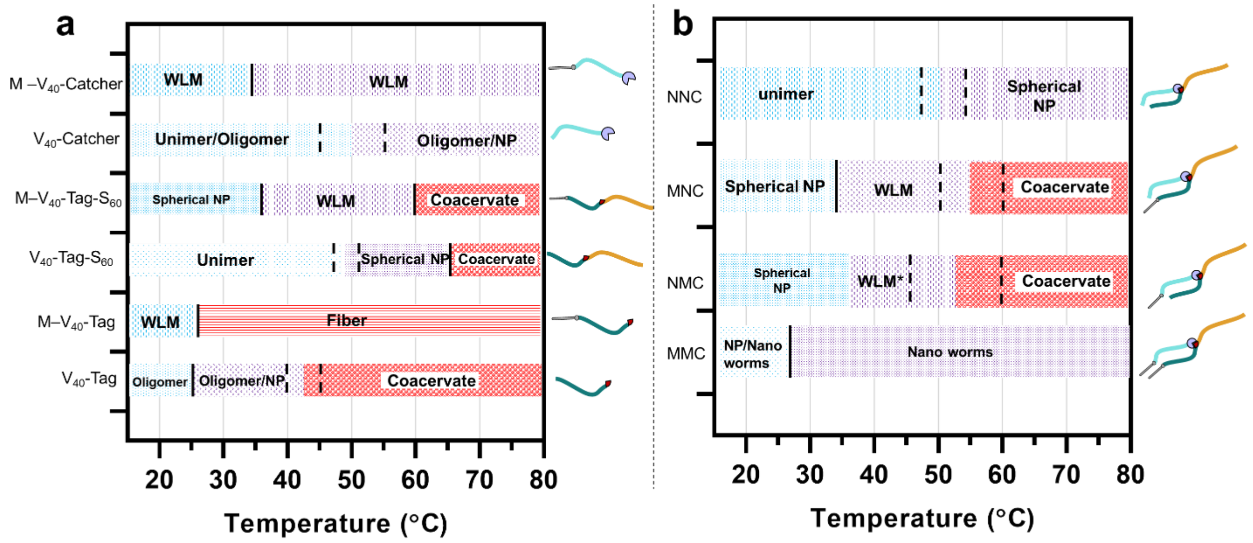

Figure S19. The schematic phase diagram for temperature-dependent nano-assembly of linear controls **(a)** and star amphiphiles **(b)** derived from the collection of turbidimetry, DLS, and TEM studies. The dashed black vertical lines show the approximate temperature range for concentration-dependent transitions in nano- or meso-assemblies. The solid vertical lines are added to denote concentration-independent transitions. The combination of dual lipidation and branching in MMC is necessary to form nanoworms over a broad window of temperature and concentration ranges. WLM is worm-like micelles. The plot in b is presented in the manuscript as Figure 6b and is included here for comparison.

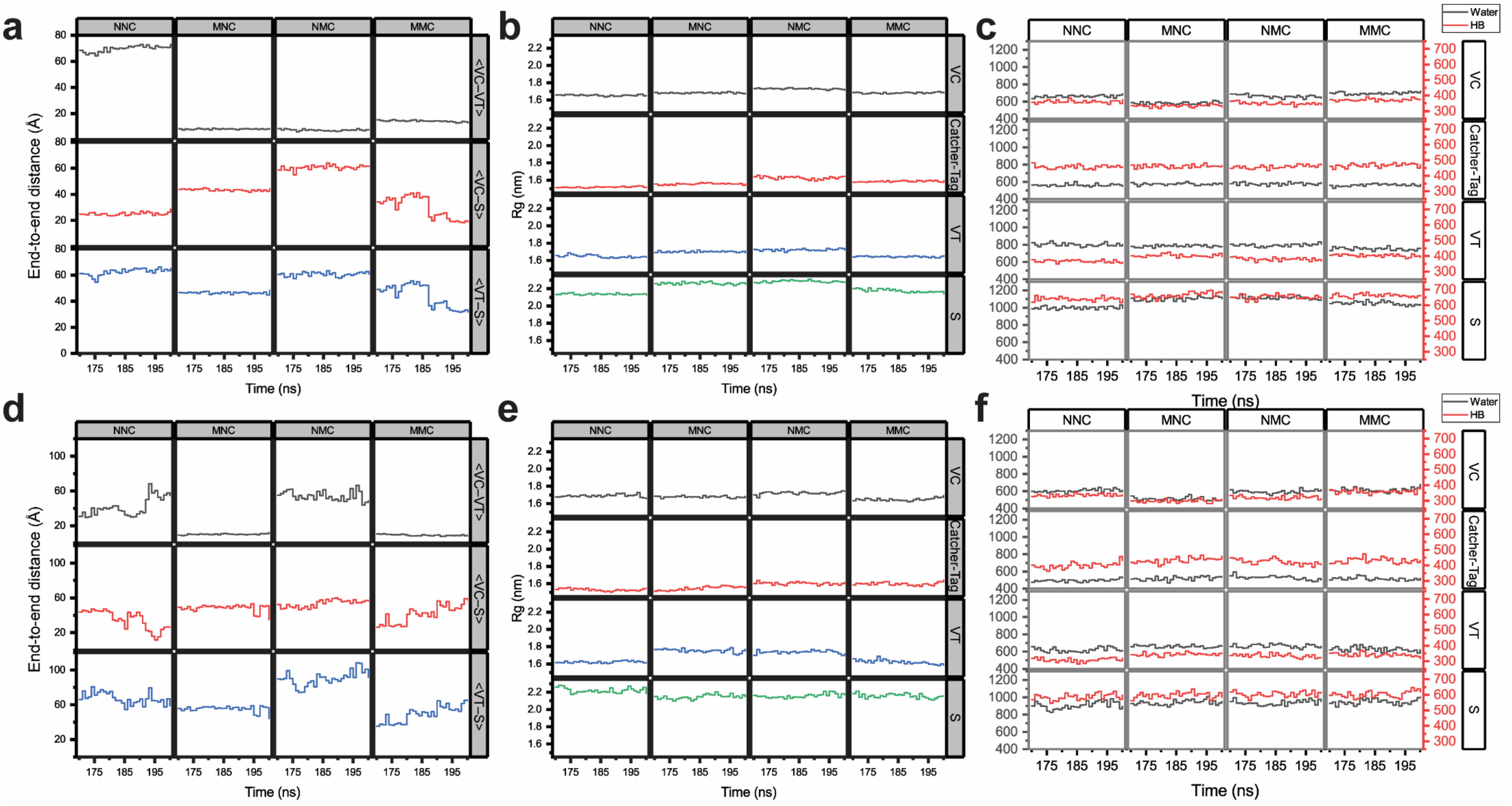

Figure S20. The trellis plots depicting the change in 15 molecular features as a function of simulation time at 5 °C **(a–c)** and 67 °C **(d–f)** for star amphiphiles. **(a,d)** The pair-wise distance between the ELP arms in angstroms. **(b,e)** The radii of gyration of different domains. **(c,f)** The average number of water molecules in the first hydration layer and the number of hydrogen bonds between water and residues in each domain. VC: The V_{40} linearly fused to the Catcher domain; VT: V40 linearly fused to the Tag domain; S: S_{60}. Catcher-Tag refers to the branching point formed after the reaction between SpyCatcher and SpyTag. Simulation data are plotted at 1 ns interval between 170–200 ns.

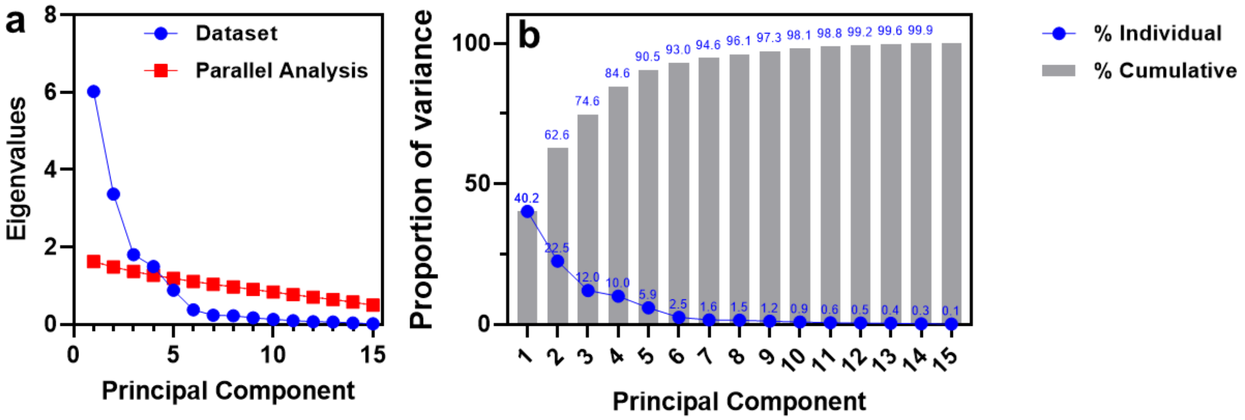

Figure S21. Component selection using parallel analysis **(a)** and proportion of variance contained within each principal component (PC) **(b)**. The first 3 PCs account for ~75% of variations in the dataset.

6. References

1. McDaniel, J. R.; Mackay, J. A.; Quiroz, F. G.; Chilkoti, A., Recursive directional ligation by plasmid reconstruction allows rapid and seamless cloning of oligomeric genes. *Biomacromolecules* **2010,** *11* (4), 944-952.

2. Luginbuhl, K. M.; Mozhdehi, D.; Dzuricky, M.; Yousefpour, P.; Huang, F. C.; Mayne, N. R.; Buehne, K. L.; Chilkoti, A., Recombinant Synthesis of Hybrid Lipid-Peptide Polymer Fusions that Self-Assemble and Encapsulate Hydrophobic Drugs. *Angew. Chem. Int. Ed. Engl.* **2017,** *56* (45), 13979-13984.

3. Hossain, M. S.; Liu, X.; Maynard, T. I.; Mozhdehi, D., Genetically Encoded Inverse Bolaamphiphiles. *Biomacromolecules* **2020,** *21* (2), 660-669.

4. Meyer, D. E.; Chilkoti, A., Purification of recombinant proteins by fusion with thermally-responsive polypeptides. *Nat. Biotechnol.* **1999,** *17* (11), 1112-1115.

5. Hagan, R. M.; Bjornsson, R.; McMahon, S. A.; Schomburg, B.; Braithwaite, V.; Buhl, M.; Naismith, J. H.; Schwarz-Linek, U., NMR Spectroscopic and Theoretical Analysis of a Spontaneously Formed Lys-Asp Isopeptide Bond. *Angew. Chem. Int. Edit.* **2010,** *49* (45), 8421-8425.
